# Excavating important nodes in complex networks based on the heat conduction model

**DOI:** 10.1038/s41598-024-58320-3

**Published:** 2024-04-02

**Authors:** Haifeng Hu, Junhui Zheng, Wentao Hu, Feifei Wang, Guan Wang, Jiangwei Zhao, Liugen Wang

**Affiliations:** 1 Pingdingshan University, Pingdingshan, 467000 China; 2 China PingMei ShenMa Group, Pingdingshan, 467099 China

**Keywords:** Heat conduction model, Degree density, Network density, Distance, SIR model, IC model, Communication and replication, Computational models

## Abstract

Analyzing the important nodes of complex systems by complex network theory can effectively solve the scientific bottlenecks in various aspects of these systems, and how to excavate important nodes has become a hot topic in complex network research. This paper proposes an algorithm for excavating important nodes based on the heat conduction model (HCM), which measures the importance of nodes by their output capacity. The number and importance of a node’s neighbors are first used to determine its own capacity, its output capacity is then calculated based on the HCM while considering the network density, distance between nodes, and degree density of other nodes. The importance of the node is finally measured by the magnitude of the output capacity. The similarity experiments of node importance, sorting and comparison experiments of important nodes, and capability experiments of multi-node infection are conducted in nine real networks using the Susceptible-Infected-Removed model as the evaluation criteria. Further, capability experiments of multi-node infection are conducted using the Independent cascade model. The effectiveness of the HCM is demonstrated through a comparison with eight other algorithms for excavating important nodes.

## Introduction

In the real world, the phenomenon of networks has a very broad application, and complex systems with numerous entities can be represented as networks^1^. A complex network can be thought of as the abstract representation of a complex system^2^, where the nodes represent the entities in the system and the edges represent the relationships between them. In computer networks, computers can be abstracted as nodes, and the network cables between computers can be abstracted as edges. In social networks, people can be abstracted as nodes, and the relationships between them can be abstracted as edges. In a complex network, a small number of nodes that play a key role in its structure and operation are called important nodes. The protection and utilization of important nodes can ensure the security and functional effectiveness of the complex system. In computer networks, redundant backup for the links to important equipment can provide additional security for network communication. In social networks, the news posted by important people spreads faster. In biological networks, important nodes play an essential role in disease discovery and drug development. Excavating important nodes is therefore crucial for various real-world applications.

In recent decades, many achievements have emerged in the study of important node excavation, and the results can be classified into node-based, edge-based, and node-edge fusion algorithms. The better-known node-based algorithms are the degree centrality (DC)^3^ and K-shell^4^. The DC measures the importance of a node by the number of neighbor nodes^5^, which only considers the most local information. It is fast in computation but poor in accuracy. The information of second-order or third-order neighbor nodes is further integrated to improve the accuracy^6^, which, however, increases the time complexity. The K-shell measures the importance of nodes by their location information in the network, and recursively deletes nodes with the same degree value. The greater the degree value used to delete a node, the more important the node is^7^. The K-shell only considers the degree of a node. It is fast in computation speed, but the results are much more coarse-grained. The classical edge-based algorithms are the betweenness centrality (BC)^8^ and closeness centrality (CC)^9^. In BC, the greater the number of shortest paths that pass through a node are, the more important the node is, whereas in CC, the fewer edges that a node passes through to other nodes, the more important the node is. These two algorithms both introduce global information while being less computation-efficient. Scholars also integrate the attributes of nodes and edges to excavate important nodes. New algorithms include identification of nodes influence based on global structure model (GSM)^10^, identification of nodes influence based on Global Structure Influence(GSI)^11^, k-shell based key node recognition method (KBKNR)^12^, influential node identification by aggregating local structure information (ALSI)^1^, and others. GSM calculates the importance of nodes through the K-shell values and shortest paths, and it considers that importance is proportional to the K-shell value and inversely proportional to the length of the shortest path. GSI considers that the degree and K-shell value have a great relationship with network structure and uses them to determine the importance of nodes while also integrating the number of nodes. KBKNR improves the K-shell algorithm by differentiating nodes in the same layer through neighbor nodes and second-order neighbor nodes based on the K-shell hierarchy, which makes the K-shell more refined. ALSI uses different formulas to calculate the importance of nodes by comparing their K-shell values, and it considers that the own degree, neighbor degree, and K-shell values determine the importance of nodes^13^. Drawing inspiration from real-world physics formulas, researchers have proposed the gravity model and continuously made improvements. Recently, researchers have addressed the issue of only focusing on the local static geographical distances between nodes and neglecting the dynamic interactions between nodes in real networks. They have introduced the Effective Distance Gravity Model^14^, which considers both global and local information of complex networks. By utilizing effective distance to merge static and dynamic information^15^, this method can uncover hidden topological structures in real-world networks and obtain more accurate results. To tackle the problem of the gravity model ignoring the surrounding environment of nodes, researchers have proposed a method based on an adaptive truncation radius and omni-channel paths^16^. This method integrates multiple node attributes and accurately describes the distance of node interactions, demonstrating good stability on networks with different scales and structural features. These studies have provided valuable insights for the development of this work.

Real-world networks have numerous stochastic characteristics. The importance of different nodes is closely related to the characteristics of the network, and excavating important nodes through multiple attributes is much more efficient than through a single attribute^17^. This is the basis for this paper’s consideration of the importance of nodes from the perspective of how much contribution they make using nodes, edges, and structural characteristics as indicators in virtue of the heat conduction model (HCM). The fundamental concepts of the HCM are described in the sequel.

### Basic idea

Person A interacts socially with person B, who may be Person A’s colleague, superior, or friend. Who among these is the most important? For A, who helps A more is more important. In other words, the more help A provides, the more important A is perceived^18^. The amount of help provided is influenced by various factors. Firstly, the more resources A possesses, the more help A is likely to offer^19^. Secondly, the amount of help provided also depends on the ability gap between A and B^20^. If person A has greater capabilities than B, A can offer more assistance than B^21,22^. Thirdly, the closer the relationship between A and B, the more A is willing to help B^23^. Fourthly, People who are directly known by A are more likely to accept A's help than those who are indirectly known^24,25^. Fifthly, the greater the influence of B is, the more A is motivated to help, as A may need B's help in the future^26–28^. These factors provide new idea for excavating important nodes in network analysis. Based on these five influencing factors, this paper evaluates the importance of nodes in complex networks by considering five indicators: degree, eigenvector centrality, distance, network density, and degree density. By leveraging a heat conduction model from the real world, the paper calculates the output capacity of nodes. The larger the values are, the more important the nodes are. The main contributions of this paper are summarized as follows^29^:

1. A new algorithm for excavating important nodes, the HCM, is proposed. This algorithm measures the importance of nodes from the perspective of how much contribution the nodes provide to other nodes. In other words, the importance of nodes is measured by their output capacity in complex networks.
2. The factor of the difference between nodes is considered to determine their output capacity values, which enhances the differentiation of output capacity and makes the evaluation of node importance more accurate. Meanwhile, the HCM is more in line with reality.
3. Real-world networks have numerous stochastic characteristics. The HCM considers the network density and the degree density of other nodes, which reduces the influence of the network structure on its accuracy and makes it more universal.

The remainder of this paper is organized as follows. Section “Preliminaries” describes the definitions involved in the HCM. In Section “Proposed algorithm”, the HCM process is specified. Simulation experiments are conducted in Section “Experimental results” and the experimental results are analyzed. Finally, Section “Conclusion” concludes this work.

## Preliminaries

The network used in this paper is an undirected unweighted network, denoted by *G*, and $G = (Vertex, Edge)$, in which *Vertex* denotes a node and *Edge* denotes an edge. In this section, the concepts and theoretical models are described.

1. HCM: This model describes the process of heat conduction in a solid, and is an algorithm for calculating the value of heat conducting from a high-temperature object to a low-temperature object. The HCM is defined as follows:
  1 $$Q = \frac{\Delta T * K * A}{{\Delta L}}$$
  where *Q* is the value of conducted heat, *ΔT* is the temperature difference between the objects, *K* is the coefficient of heat conduction, *ΔL* is the distance traveled, and *A* is the contact area between the objects.
2. DC: *G* is denoted by the adjacency matrix *A* = (*a*_*ij*_)N*N, and the value *a*_*ij*_ is located in the jth column and the ith row of the matrix *A*^30^. When *a*_*ij*_ = 1, there is an edge between nodes *v*_*i*_ and *v*_*j*_, while *a*_*ij*_ = 0 indicates that there is no edge between them. The degree of node *v*_*i*_ is defined as follows:
  2 $$D\left( {vi} \right) = \sum\limits_{j = 1}^{N} {aij} = \sum\limits_{i = 1}^{N} {aji}$$
  where *v*_*i*_ denotes the node number, *D*(*v*_*i*_) denotes the degree value of node *v*_*i*_, and *N* denotes the number of nodes.
  To facilitate the degree of nodes in different networks, the degree values are normalized^5^. DC is defined as follows:
  3 $$DC\left( {vi} \right) = \frac{{D\left( {vi} \right)}}{N - 1}$$
3. Eigenvector centrality (EC)^31^: *G* is denoted by the adjacency matrix *A* = (*a*_*ij*_)N*N, and *A* is a square matrix of dimensions *N* × *N*. An eigenvalue $\lambda i$ of the square matrix *A* is a scalar, and the corresponding eigenvector $xi$ is a non-zero vector, which satisfies the following relationship:
  4 $$A * xi = \lambda i * xi$$
  Therefore,
  5 $$xi = \frac{1}{\lambda i} * A * xi = \frac{1}{\lambda i} * \sum\limits_{j = 1}^{N} {aij * xj}$$
  In general, there are multiple eigenvalues $\lambda$ satisfying Eq. (4), as well as multiple corresponding eigenvectors $x$. When $\lambda$ takes the maximum value ${\text{max}}\lambda$, the obtained eigenvector $\max x$ is an important eigenvector. EC is defined as follows:
  6 $$EC\left( {vi} \right) = \frac{1}{\max \lambda } * \sum\limits_{j = 1}^{N} {aij * xj}$$
4. CC: Node *v*_*i*_ of *G* is connected to *v*_*j*_, then there is at least one path $path\left( {vi,vj} \right)$ between nodes *v*_*i*_ and *v*_*j*_, and the path containing the least number of edges is the shortest path $spath\left( {vi,vj} \right)$. The distance $R\left( {vi,vj} \right)$ between nodes *v*_*i*_ and *v*_*j*_ is defined as^32^:
  7 $$R\left( {vi,vj} \right) = \left| {spath\left( {vi,vj} \right)} \right|$$
  where $\left| {spath\left( {vi,vj} \right)} \right|$ is the number of edges that the shortest path contains.
  The smaller the distance between a node and other nodes, the closer it is to the network center. CC is defined as:
  8 $$CC\left( {vi} \right) = \frac{N - 1}{{\sum\limits_{j = 1}^{N} {R\left( {vi,vj} \right)} }}$$
5. Network density: Network density measures the closeness of the connections between nodes^33^. A larger value indicates that nodes are more closely connected, while a smaller value indicates that nodes are more loosely connected. Its definition is:
  9 $$Density\left( G \right) = \frac{{2 * \left| {Edge} \right|}}{{N * \left( {N - 1} \right)}}$$
  where $\left| {Edge} \right|$ denotes the actual number of edges and *N* is the number of nodes.
6. Degree density: The area of a circle is calculated by taking the node *v*_*j*_ as the center of the circle and the distance $R\left( {vj,vi} \right)$ as the radius. The ratio of $D\left( {vj} \right)$ to the area is called degree density from *v*_*j*_ to *v*_*i*_, and is defined as:
  10 $$Dd\left( {vi,vj} \right) = \frac{{D\left( {vj} \right)}}{{\pi * R\left( {vi,vj} \right)^{2} }}$$

The degree density is used to adjust the influence of the receiving node on the output node.

## Proposed algorithm

The HCM incorporates five factors. Eigenvector centrality is used to calculate the feature vector values for each node, while degree centrality is used to calculate the degree values for each node. The degree values are then used as coefficients, and the difference in feature vector values between two nodes is considered as the temperature difference *ΔT*. The greater the degree, the more the output value; The larger the difference between the eigenvectors, the larger the output value^34^. The network density is used as the thermal conductivity coefficient *K*, the higher the network density, the closer the connection between nodes, and the larger the output value^35^. The degree density from a node to the target node is considered as the contact area *A*, the higher the degree density of the acceptance node, the higher the influence, and the higher the output value. The distance between two nodes is used to calculate *ΔL*, the greater the distance, the smaller the output value^36^. With the help of a heat conduction model formula, the value of *Q* is calculated as the output value for the target node. With Eq. (1), the output value of node *v*_*i*_ for *v*_*j*_ is defined as follows:

11 $$Q\left( {vi,vj} \right) = \frac{{D\left( {vi} \right) * e^{{EC\left( {vi} \right) - EC\left( {vj} \right)}} * Density\left( G \right) * Dd\left( {vi,vj} \right)}}{{R\left( {vi,vj} \right)}}$$

In this algorithm, the output capacity of nodes is measured by their output value. As the number of nodes in different networks is different, the output value is normalized. The output capacity of *v*_*i*_ is defined as:

12 $$I\left( {vi} \right) = \frac{1}{N - 1} * \sum\limits_{j = 1\& j \ne i}^{N} {Q\left( {vi,vj} \right)}$$

### Algorithm process description

First, the capacity difference between nodes is calculated by their degree and eigenvector values. The network density, the degree density, and the distance between nodes are then computed. Finally, the output value is calculated using the HCM formula. The pseudo-code for this algorithm is shown in Table 1.

**Table 1:**
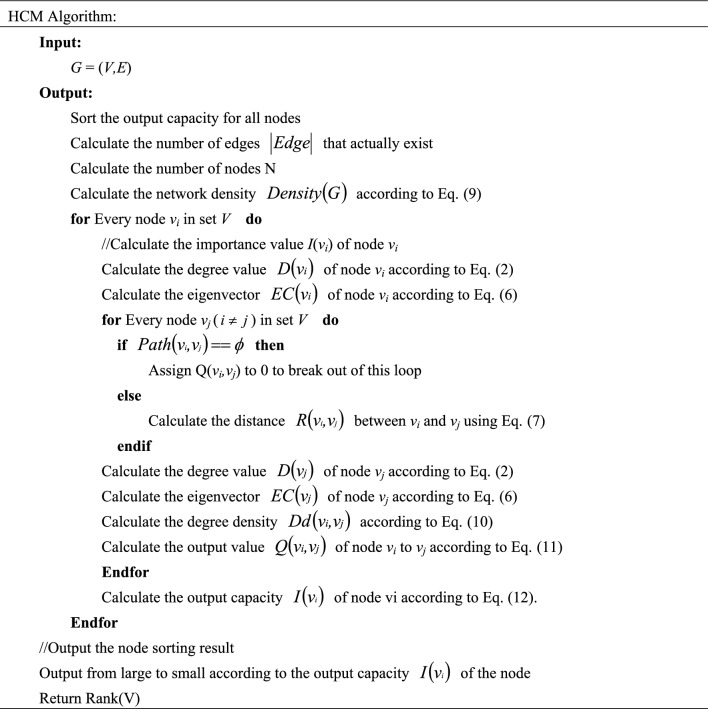
Pseudo-code of the HCM algorithm.

### Example description

**Figure 1:**
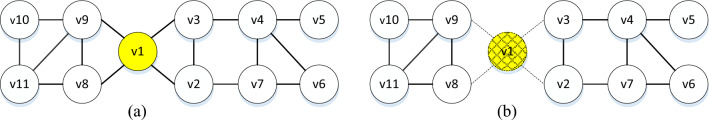
An example of a network. (**a**) is the original diagram, and (**b**) is the comparison diagram after processing. The network consists of 11 nodes and 16 edges, and the yellow node is an example node.

In Fig. 1a, node *v*_*1*_ plays an important role in the topology of the entire network. If node *v*_*1*_ is removed, as shown in Fig. 1b, the entire network becomes two disconnected sub-nets.

Therefore, node *v*_*1*_ is an important node in the network shown in Fig. 1a.

1. Calculate the degree value, the eigenvector value, and the distance between nodes.
  Figure 1a is taken as an example to illustrate the calculation process of the HCM. The degree value of each node, the eigenvector value, and the distance between each node are first determined. The results are shown in Table 2.

**Table 2:** The degree value, the eigenvector value, and the distance between nodes.

Node	*D*(*v*_*i*_)	*EC*(*v*_*i*_)	*CC*(*v*_*i*_)	$R\left( {vi,vj} \right)$										
				*v*_*1*_	*v*_*2*_	*v*_*3*_	*v*_*4*_	*v*_*5*_	*v*_*6*_	*v*_*7*_	*v*_*8*_	*v*_*9*_	*v*_*10*_	*v*_*11*_
*v*_*1*_	4	0.44507	0.55556	*Ø*	1	1	2	3	3	2	1	1	2	2
*v*_*2*_	3	0.29872	0.5	1	*Ø*	1	2	3	2	1	2	2	3	3
*v*_*3*_	3	0.30294	0.52632	1	1	*Ø*	1	2	2	2	2	2	3	3
*v*_*4*_	4	0.22330	0.45455	2	2	1	*Ø*	1	1	1	3	3	4	4
*v*_*5*_	1	0.06995	0.32258	3	3	2	1	*Ø*	2	2	4	4	5	5
*v*_*6*_	2	0.13435	0.34483	3	2	2	1	2	*Ø*	1	4	4	5	5
*v*_*7*_	3	0.20561	0.43478	2	1	2	1	2	1	*Ø*	3	3	4	4
*v*_*8*_	3	0.38080	0.43478	1	2	2	3	4	4	3	*Ø*	1	2	1
*v*_*9*_	4	0.43838	0.45455	1	2	2	3	4	4	3	1	*Ø*	1	1
*v*_*10*_	2	0.24139	0.33333	2	3	3	4	5	5	4	2	1	*Ø*	1
*v*_*11*_	3	0.33222	0.34483	2	3	3	4	3	5	5	1	1	1	*Ø*

Ø indicates that there is no edge between nodes. From Table 1, the maximum degree value in the example is 4, and the maximum distance between nodes is 5.
2. Calculate the network density.
  The network density can be determined according to Eq. (9): *Density*(*G*) = 0.29091.
3. Calculate the degree density.
  The degree density from each node to *v*_*1*_ is computed according to Eq. (10) and the results are shown in Table 3.

**Table 3:** The degree density from each node to *v*_*1*_.

*Dd* (*v*_*2*_,*v*_*1*_)	*Dd* (*v*_*3*_,*v*_*1*_)	*Dd* (*v*_*4*_,*v*_*1*_)	*Dd* (*v*_*5*_,*v*_*1*_)	*Dd* (*v*_*6*_,*v*_*1*_)	*Dd* (*v*_*7*_,*v*_*1*_)	*Dd* (*v*_*8*_,*v*_*1*_)	*Dd* (*v*_*9*_,*v*_*1*_)	*Dd* (*v*_*10*_,*v*_*1*_)	*Dd* (*v*_*11*_,*v*_*1*_)
0.95493	0.95493	0.31831	0.03537	0.07074	0.23873	0.95493	1.27324	0.15915	0.23873
4. Output value of node *v*_*1*_.

The output value of *v*_*1*_ for each node is calculated according to Eq. (11), and the results are shown in Table 4.

**Table 4:** The output value of *v*_*1*_ for each node.

*Q* (*v*_*1*_,*v*_*2*_)	*Q* (*v*_*1*_,*v*_*3*_)	*Q* (*v*_*1*_,*v*_*4*_)	*Q* (*v*_*1*_,*v*_*5*_)	*Q* (*v*_*1*_,*v*_*6*_)	*Q* (*v*_*1*_,*v*_*7*_)	*Q* (*v*_*1*_,*v*_*8*_)	*Q* (*v*_*1*_,*v*_*9*_)	*Q* (*v*_*1*_,*v*_*10*_)	*Q* (*v*_*1*_,*v*_*11*_)
1.28632	1.28091	0.29435	0.01820	0.03414	0.22470	1.18495	1.49154	0.14454	0.19798

The average value of the output values from *v*_*1*_ to all other nodes is calculated using Eq. (12) to measure the output capacity of *v*_*1*_. I(***v***_***1***_) = 0.615762.

According to the above calculation process, the output capacity of each node is calculated and then sorted in descending order. The sorting results are shown in Table 5.

**Table 5:** The output capacity of each node.

*I* (*v*_*1*_)	*I* (*v*_*9*_)	*I* (*v*_*4*_)	*I* (*v*_*3*_)	*I* (*v*_*8*_)	*I* (*v*_*2*_)	*I* (*v*_*7*_)	*I* (*v*_*11*_)	*I* (*v*_*6*_)	*I* (*v*_*10*_)	*I* (*v*_*5*_)
0.61576	0.53142	0.38379	0.36353	0.34412	0.33343	0.28214	0.26374	0.14042	0.13362	0.04395

As can be seen from Table 5, node *v*_*1*_ has the highest output capacity, so it is the most important node, followed by *v*_*9*_ and *v*_*4*_. It can be seen from Table 2 that the degree values of nodes *v*_*1*_, *v*_*4*_*,* and *v*_*9*_ are all 4. The values of *EC*(*v*_*1*_) and *EC*(*v*_*9*_) differ only slightly, but the values of *CC*(*v*_*1*_) and *CC*(*v*_*9*_) show a significant difference. This indicates that node *v*_*1*_ is closer to the network center. Therefore, the output capacity of node *v*_*1*_ is stronger. The values of *CC*(*v*_*4*_) and *CC*(*v*_*9*_) are the same, but the difference between *EC*(*v*_*4*_) and *EC*(*v*_*9*_) values is significant, so the output capacity of node *v*_*9*_ is stronger. Due to the consideration of many influencing factors, the HCM can distinguish the output capacity of nodes.

### Time complexity analysis

The HCM algorithm consists of four stages, and the temporal complexity analysis results are described below. In the first stage, calculating the number of edges and nodes in the network has time complexities of O(ǀEdgeǀ) and O(N), respectively. In the second stage, using the Dijkstra method to calculate the distance between any two nodes in the network has a time complexity of O(N^2^). In the third stage, calculating the degree, eigenvector centrality, and degree density of each node has time complexities of O(N < d >), O(N^2^), and O(N), respectively. In the fourth stage, outputting the nodes in descending order of their importance has a time complexity of O(N^2^). In summary, the HCM algorithm has a time complexity of O(N^2^).

## Experimental results

### Evaluation index

1. SIR infectious disease model^37^.
  The SIR model is a mathematical model applied to information transmission research, and an essential standard for evaluating important nodes in complex networks^38^. The SIR model splits the population into susceptible, infective, and removed categories, with the respective numbers of all populations at time t denoted by S(t), I(t), and R(t)^39^. In disease transmission, the susceptible population becomes the infective population with infection probability *α*, and the infective population turns into the removed population with recovery probability *β*. The mathematical model of the SIR is defined as follows:
  13 $$\left\{ {\begin{array}{*{20}l} {S\left( {\Delta t} \right) = - S\left( t \right) * \alpha * \Delta t} \hfill \\ {I\left( {\Delta t} \right) = S\left( t \right) * \alpha * \Delta t - I\left( t \right) * \beta * \Delta t} \hfill \\ {R\left( {\Delta t} \right) = I\left( t \right) * \beta * \Delta t} \hfill \\ \end{array} } \right.$$
  where *Δ*t represents the time interval.
  In the experiment, one node is selected as the infective node, and the others are chosen to be the susceptible nodes. Infective nodes infect all susceptible nodes with probability *α*. The number of susceptible nodes that turned into infective nodes is used as the infection value to measure the infection capacity of nodes.
2. IC model^40^.
  The independent cascade model is an information propagation model that provides an abstract description of the process by which information spreads. In this model, a node is designated as a seed node, and each edge in the network is assigned a propagation probability denoted as "P". The seed node attempts to activate its neighboring nodes with a probability of "P"^41^. Each node has only one opportunity to activate another node, and if it fails, it will not make any further attempts to activate that particular node^42^. This propagation process is iterated until no more nodes in the network can be activated. IC model was originally used to describe the dissemination of commodity information in marketing and has now been widely used in the analysis of influence spreading in various fields^43,44^.
3. Kendall $$\tau$$ coefficient^45^.

The Kendall $\tau$ coefficient is a statistic that measures the similarity between two sets of random numbers. First, a network can determine the infection value *s*_*i*_ of each node through the SIR model. The infection value of all nodes is a set that can be expressed as S = (*s*_*1*_*, s*_*2*_*, …, s*_*i-1*_*, s*_*i*_*, s*_*i*+*1*_*, …, s*_*n*_), with *n* being the number of nodes. The HCM calculates the output capacity set of all nodes H = (*Q*_*1*_*, Q*_*2*_*, …, Q*_*i-1*_*, Q*_*i*_*, Q*_*i*+*1*_*, …, Q*_*n*_), with *Q*_*i*_ being the output capacity of node *v*_*i*_. When *Q*_*i*_ > *Q*_*i*+*1*_, there is *s*_*i*_ > *s*_*i*+*1*_, or when *Q*_*i*_ < *Q*_*i*+*1*_, there is *s*_*i*_ < *s*_*i*+*1*_, and then the two sequences (*Q*_*i*_*, Q*_*i*+*1*_) and (*s*_*i*_*, s*_*i*+*1*_) are regarded as being similar. Otherwise, they are not considered similar. The Kendall $\tau$ coefficient is used to measure the similarity between the two groups of sequences S and H. The calculation formula is as follows^46^:

14 $$\tau (S,H) = \frac{{2 * \left( {n_{c} - n_{d} } \right)}}{n(n - 1)}$$

where *n*_*c*_ and *n*_*d*_ denote the number of similar and dissimilar sequences, respectively. Higher $\tau$ values indicate greater similarity between H and S, while lower values indicate greater dissimilarity.

### Data description

To evaluate the accuracy and applicability of the HCM, nine real networks of three sizes—large, medium, and small—are selected with details shown in Table 6.

**Table 6:** Statistical characteristics of nine actual networks.

Size	DataSets	|Vertex|	|Edge|	*Density*(G)	< *d* >	Max*d*	Category	Node meaning	Edge meaning
Small	David^47^	112	425	0.068372	7.589	49	Lexical network	Noun	Adjacency
	Netscience^48^	379	914	0.012760	4.823	34	Co-authorship network	Author	Co-authorships
Medium	Hamsterster^49^	2426	16,630	0.005654	13.71	273	Online social network	User	Friendship
	Ca-GrQc^50^	4158	13,422	0.001553	5.531	81	Collaboration network	Author	Collaboration
	AS^51^	6474	13,895	0.000663	4.293	1460	Computer network	Autonomous system	Communication
	Lastfm^52^	7624	27,806	0.000957	7.294	216	Social network	Users	Relationships
	Dblp^53^	12,591	49,635	0.000626	7.884	709	Citation network	Publication	Citation
Large	Ca-Astroph^54^	18,771	198,050	0.001124	21.102	504	Collaboration network	Papers	Collaborations
	EmailEU^51^	32,430	54,397	0.000103	3.355	623	Communication network	Person	Email

All networks data are available at https://github.com/hhf602/HCM.

### Contrast algorithm description

To verify the effectiveness of the HCM, eight algorithms for excavating important nodes are selected for comparison, including four well-known and more recent. The eight algorithms are described in Table 7.

**Table 7:** Metrics for the eight comparison algorithms.

Method	Metric index	Method type	Date
BC^8^	Number of shortest paths	Edge-based method	1977
CC^9^	Distance	Edge-based method	1966
DC^3^	Number of neighbor nodes	Node-based method	1994
EC^31^	Number and importance of neighbor nodes	Node-based method	1972
GSI^11^	K-shell, distance, degree and EC	Node-and edge-based method	2022
GSM^10^	K-shell and distance	Node-and edge-based method	2021
ALSI^1^	K-shell and degree	Node-based method	2022
KBKNR^12^	K-shell, distance and degree	Node-and edge-based method	2022

### Experimental results

To evaluate the effectiveness of the HCM more comprehensively, the probability of infection α was taken as ten values on the interval [0.01,0.1] with a step size of 0.01 in the SIR model, and the recovery probability was set to *β* =^5,10,54–57^. The experimental equipment is a desktop computer with an Intel i5-10100@3.10 Hz CPU and 32 GB memory, and the software environment is Spyder (Python 3.7.3).

1. Kendall $\tau$ value comparison.

**Figure 2:**
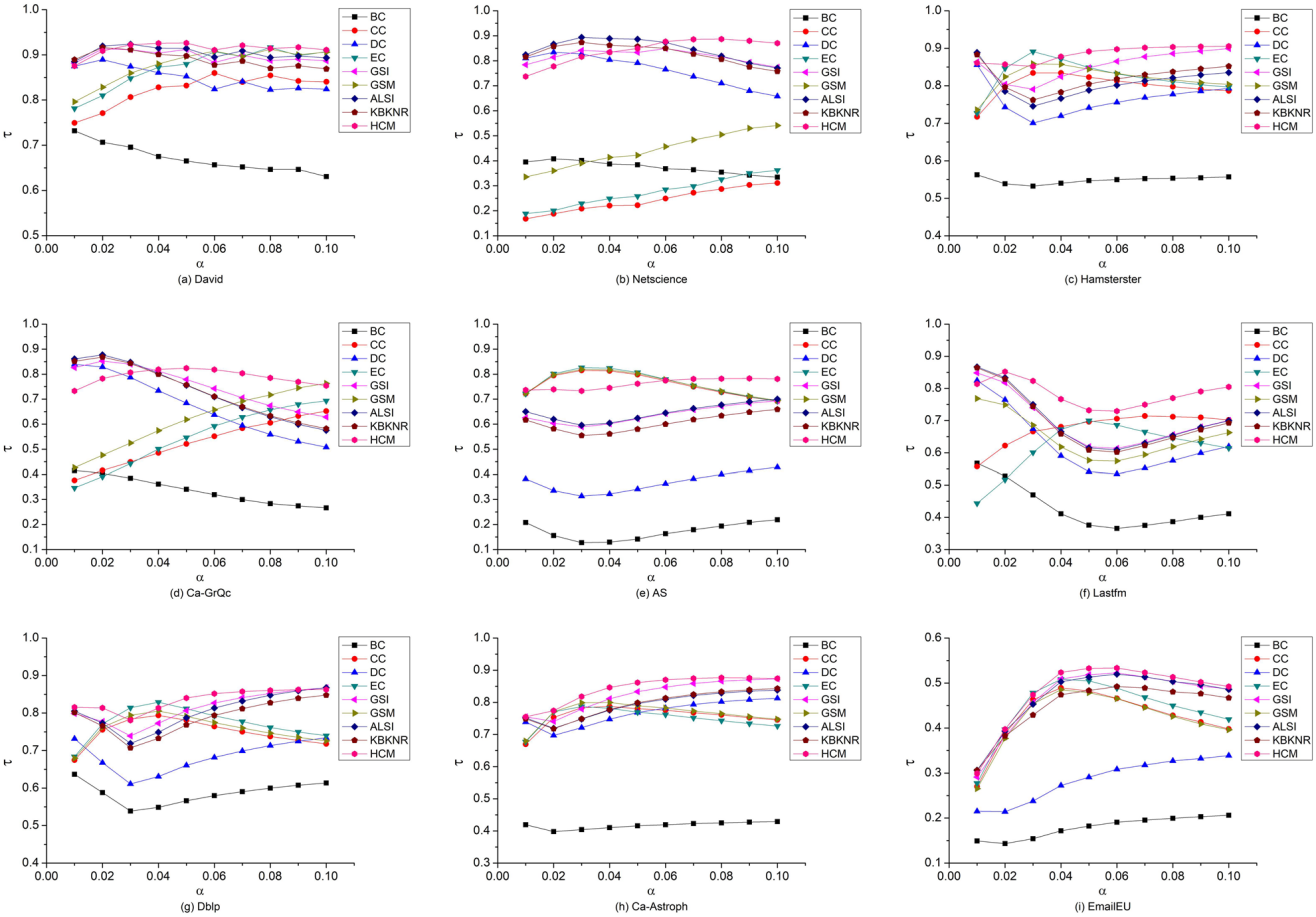
Kendall τ values of different algorithms. Among the ten τ values obtained by comparing the HCM’s calculation results with SIR, in Lastfm, Ca-Astroph, and EmailEU, nine of them are higher than those obtained by other algorithms; in Hamsterster and Dblp, eight of them are higher than other algorithms; in David and Ca-GrQc, seven of them are higher than other algorithms; and in Netscience and AS, five of them are higher than other algorithms. Generally speaking, the HCM has absolute advantages.

The output capacity calculated by nine algorithms is sorted according to node number. Similarly, the infection values calculated by the SIR model under different probabilities are sorted as well. According to Eq. (14), the sorting results of each algorithm are compared with the sorting results of the SIR model under ten probabilities, and the Kendall $\tau$ value is obtained. The comparison results are shown in Fig. 2.

To further test the performance, we compare the HCM with the other eight methods on three small actual networks. The basic statistics of these three small actual networks are summarized in Supplementary Table S10. The results (Supplementary Tables S11–S13) suggest that the HCM method are still very competitive (in-coreness performs overall best).

**Figure 3:**
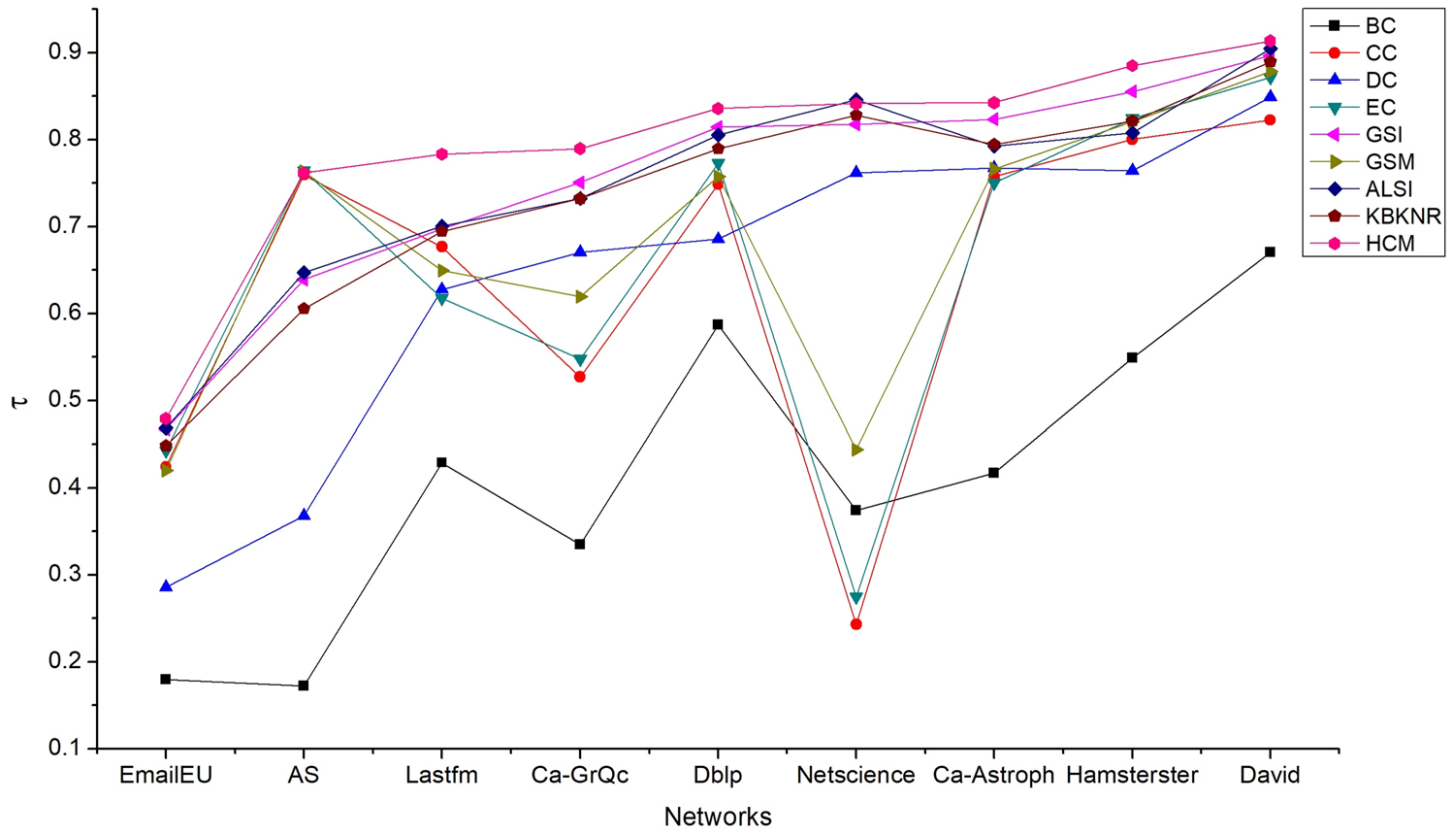
Average Kendall τ values of nine algorithms under ten infection probabilities. The average τ value of each algorithm in EmailEU is small, but the HCM is still better than the other eight algorithms. In the other eight networks, the results of the HCM are almost a horizontal line and are in the highest position. This indicates that the HCM has the best overall effect, and is suitable for various networks. Other algorithms show different performances in different networks, and the results fluctuate greatly.

In different networks above, the comparison results of the τ values obtained by each algorithm are evaluated when the infection probability α takes different values. Results show that the HCM has shown the best effect under most infection probabilities, but ordinary performance under some infection probabilities. To more comprehensively verify the effectiveness of the HCM, the average Kendall $\tau$ value obtained by various algorithms under different infection probabilities is further compared, and the results are shown in Fig. 3.

As the HCM takes into account factors such as degree, eigenvector, and distance, the effect of the algorithm is related to degree centrality, eigenvector value centrality, and closeness centrality to some extent. Netscience has a hierarchical organizational structure. From Fig. 2, the CC and EC perform the worst, resulting in a low Kendall τ value in the front part of the HCM. With the increase in their τ values, the HCM outperforms other algorithms in the interval [0.06, 0.1]. There is a big difference between the maximum degree Max *d* and the average degree < d > in EmailEU and AS. This indicates that the high degree values are concentrated on a small number of nodes, resulting in poor degree differentiation of other nodes, so the performance of the DC is poor. Influenced by DC, the τ value obtained by the HCM is relatively low. However, because the EC and CC have better performance, the HCM is still better than other algorithms. Meanwhile, by comprehensively considering the network and degree densities of other nodes, the nine networks do not significantly differ in their τ values for the HCM.

**Figure 4:**
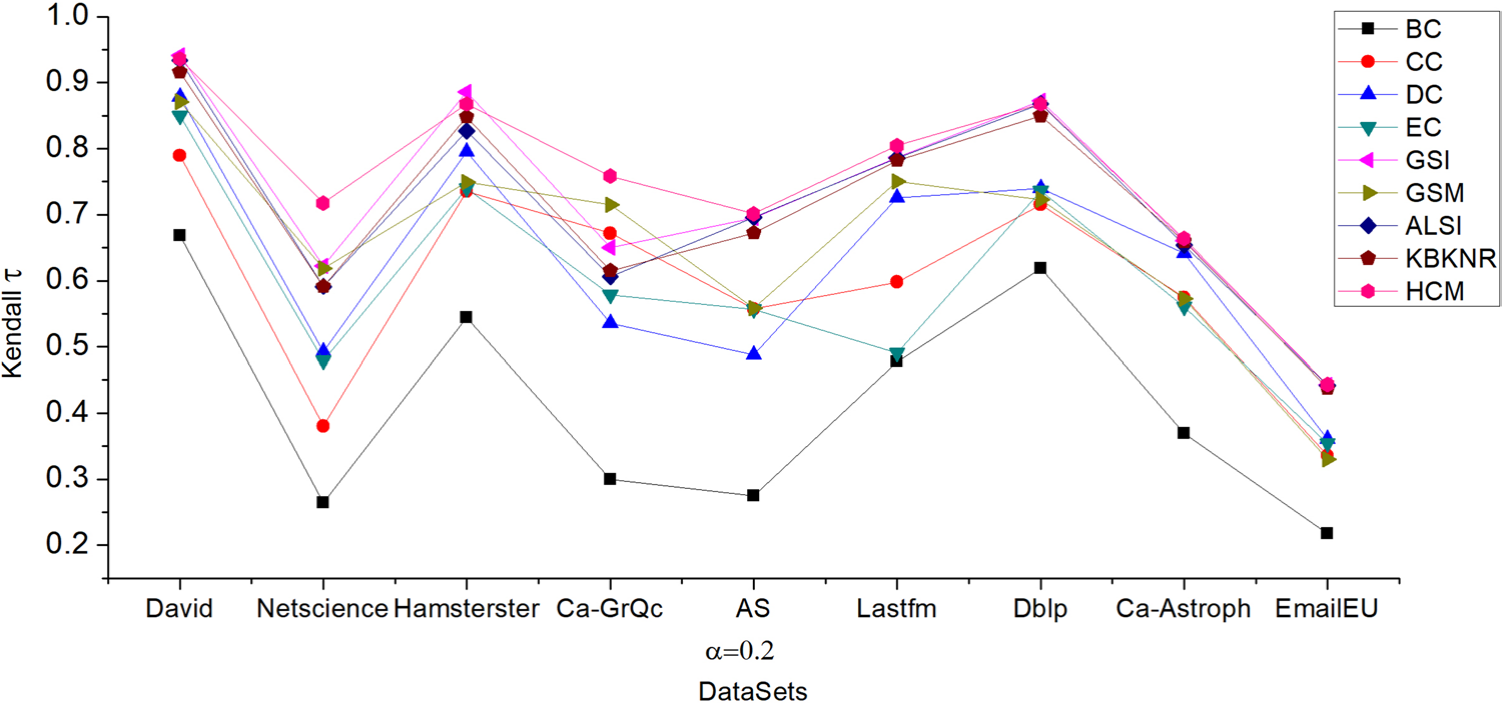
The Kendall τ between the node influence of SIR model and nine algorithms. In the six networks Netscience, Ca-GrQc, AS, Lastfm, Ca-Astroph, and EmailEU32430, the HCM obtained the highest Kendall τ value. In David, Hamsterster, Dblp, the GSI performs the best and the HCM is only marginally inferior, but the HCM's value is also greater than 0.86.

In order to accurately evaluate the effectiveness of the HCM algorithm, we increased the value of *α* in the SIR model, setting *α* = 0.2 and keeping *β* = 1, and conducted the experiment again. The comparison results of the Kendall values at *α* = 0.2 are shown in Fig. 4. The experimental results indicate that our proposed model (HCM) still performs well.

(2) Sorting comparison of node importance.

In this section, nodes are sorted in descending order according to output capability, and their positions in the sequence are compared with those of SIR. From networks of three scales, each one is selected to present David, AS, and EmailEU, respectively. In order not to lose generality, *α* of the SIR model is set to 0.04 in David and AS and 0.01 in EmailEU, while *β* is 1. To display the results more intuitively, the top ten important nodes are selected for comparison.

The top ten important nodes of each algorithm excavated in David are shown in Tables 8, 9, 10.

**Table 8:** The first ten nodes of each algorithm in David.

BC	CC	DC	EC	GSI	GSM	ALSI	KBKNR	HCM	SIR
18	18	18	18	18	18	18	18	18	18
3	3	3	3	3	3	3	3	3	3
44	52	52	52	52	52	52	52	52	52
52	44	44	44	44	44	44	44	44	44
9	28	105	105	105	105	105	105	105	105
80	105	9	51	9	28	9	9	9	9
105	9	25	25	28	25	25	28	25	25
28	27	28	26	25	51	51	25	51	28
1	25	51	55	51	9	28	51	28	51
29	26	1	32	26	26	26	26	26	26

**Table 9:** The first ten nodes of each algorithm in AS.

BC	CC	DC	EC	GSI	GSM	ALSI	KBKNR	HCM	SIR
2	2	2	2	2	2	2	2	2	2
7	10	10	10	10	10	10	10	10	10
10	7	7	7	7	7	7	7	7	7
29	3	8	1	8	1	8	8	1	1
8	1	1	8	1	3	1	1	8	8
1	4	3	3	3	8	3	3	3	3
3	6	23	4	23	4	23	23	23	4
394	8	42	23	42	6	42	42	42	6
403	29	29	6	29	29	29	29	29	23
6	22	518	42	6	5	518	6	6	5

**Table 10:** The first ten nodes of each algorithm in EmailEU.

BC	CC	DC	EC	GSI	GSM	ALSI	KBKNR	HCM	SIR
5	622	102	486	102	122	102	102	102	486
622	322	5	162	5	154	5	5	486	122
554	162	122	622	122	102	122	122	5	102
102	486	486	322	486	387	486	486	122	5
387	387	55	882	83	512	83	55	882	322
486	554	83	102	55	554	296	83	678	83
512	5	525	625	154	678	512	525	512	162
322	698	115	122	512	486	154	115	154	512
162	882	45	698	678	5	678	45	162	678
122	625	296	678	296	625	214	154	55	154

From Table 8, the important nodes excavated by GSI, GSM, ALSI, KBKNR, and HCM are completely consistent with that of the SIR model. The order of the first seven nodes of HCM and ALSI as well as the first six nodes of GSI and KBKNR is consistent with that of the SIR model. Therefore, HCM and ALSI have the best effect in excavating important nodes. Table 9 shows that nine of the top ten important nodes of GSM and EC are the same as those of the SIR model, which indicates that they have the best effect. The first eight nodes of HCM, KBKNR, and GSI are the same as those of the SIR model, while only the first six nodes of HCM are in the same order as those of SIR. HCM is less effective than GSM and EC in excavating important nodes, but better than other algorithms. It can be observed in Table 10 that eight of the first ten nodes of HCM, GSI, and ALSI are the same as those of the SIR model, and the results of excavating important nodes are the best. Followed by BC and GSM with seven nodes being the same as those of SIR. CC and DC exhibit the worst performance with only four nodes being the same as those of SIR.

**Figure 5:**
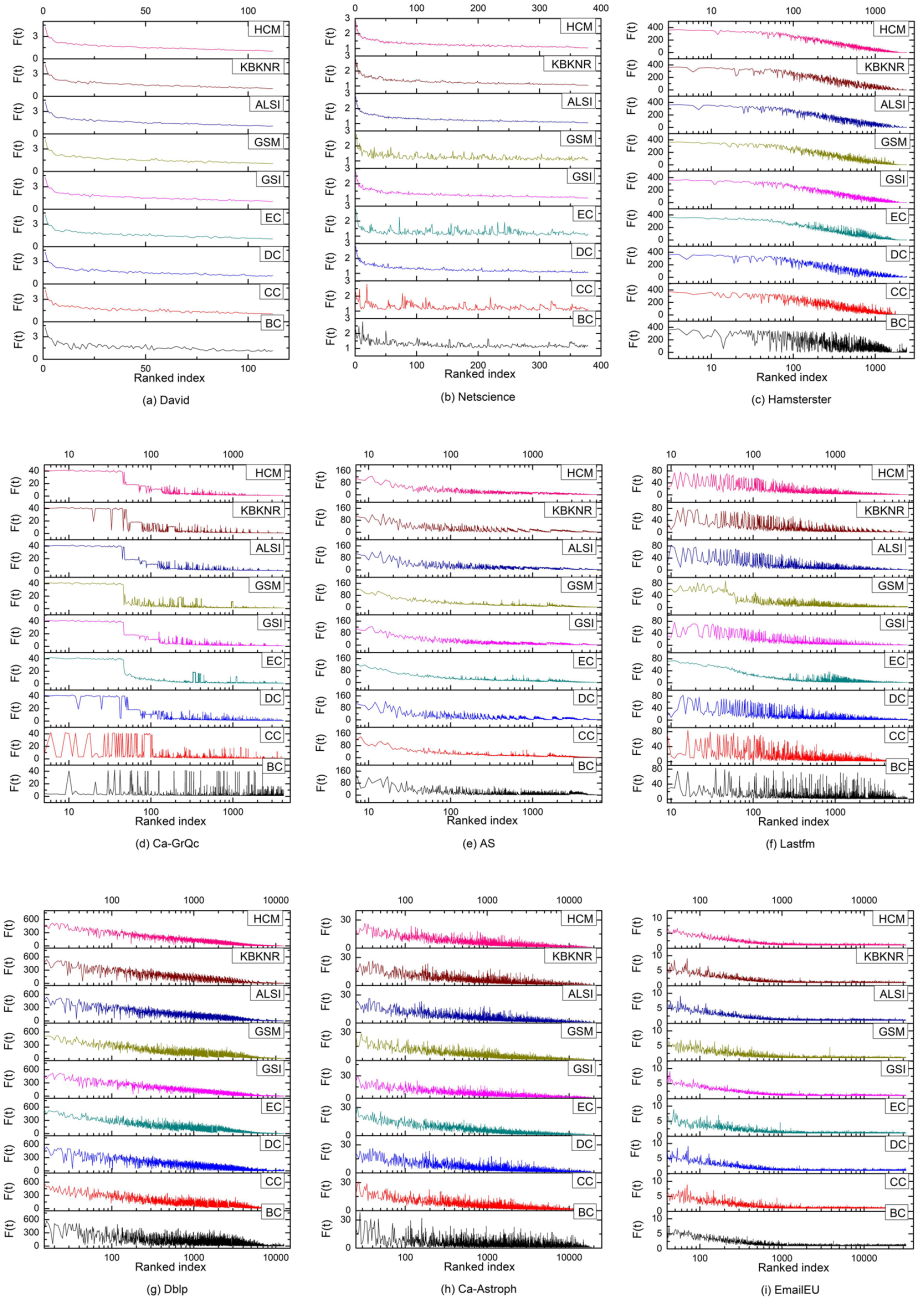
Results of re-sorting infection values by each algorithm. The x-axis represents the number of nodes in the sorting results of each algorithm. For example, 10 represents the top ten nodes with the highest output capacity. The y-axis is the infection value of each node obtained in the SIR model.

To further verify the effectiveness of the HCM in excavating important nodes, the sorting results of all nodes by various algorithms are compared with those of the SIR model. For comparability, the infection value in the SIR model is used as the reference. The infection value of each node is first obtained through the SIR model. The infection values are then re-sorted according to the node order obtained by each algorithm. When the sorting results of each algorithm are consistent with the SIR model, the new sequence of infection values is from large to small and form a smooth downward curve from left to right in the graph. To highlight the important nodes, they are presented linearly on small-scale networks like David and Netscience and presented in Log10 in other networks. To ensure the accuracy of the results, the SIR model is applied with 100 iterations in the large-scale network EmailEU and with 1000 iterations in other networks, and the average value is taken as the infection value of nodes. The results of nodes sorted by various algorithms and the SIR model are compared in Fig. 5.

As seen in Fig. 5, the curve formed by the HCM has a narrower overall fluctuation range in David, Netscience, Hamsterster, Dblp, and EmailEU in comparison to other algorithms. This indicates that the HCM is the most consistent with the SIR model in sorting the importance of nodes. In Ca-GrQc, the HCM, ALSI, GSM, GSI, and EC curves are smooth on the left, indicating that the sorting of the most important nodes by these five algorithms is consistent with that of the SIR model. They each have a burr on the right side, indicating that the sorting position of some individual nodes is different from that of the SIR model. Among them, EC exhibits the best effect with relatively less burr. In AS, the results of GSM, EC, and CC compared with the SIR model form a smooth downward curve. Although there are burrs, their number is small, and their amplitude is small as well. So, these three algorithms have the best effect. The effects of HCM and GSI are worse than those of GSM, EC, and CC, but better than those of BC, DC, KBKNR, and ALSI. In Lastfm, the left part of the curve formed by EC shows a smooth downward trend. The curve formed by GSI has burrs with a large amplitude, but their number is small. The number of burrs in the curve formed by GSM is large, but their amplitude is small. The left part of the curve formed by HCM fluctuates greatly, but the right part fluctuates less. Therefore, EC has the best effect, followed by GSI and GSM. Although HCM has no obvious advantages, it is still better than other algorithms. In Ca-Astroph, the performance of other algorithms is similar except for the BC. Based on the impact of each of the nine algorithms, the HCM has the best overall performance.

(3) Comparison of infection capacity of the top ten nodes

**Figure 6:**
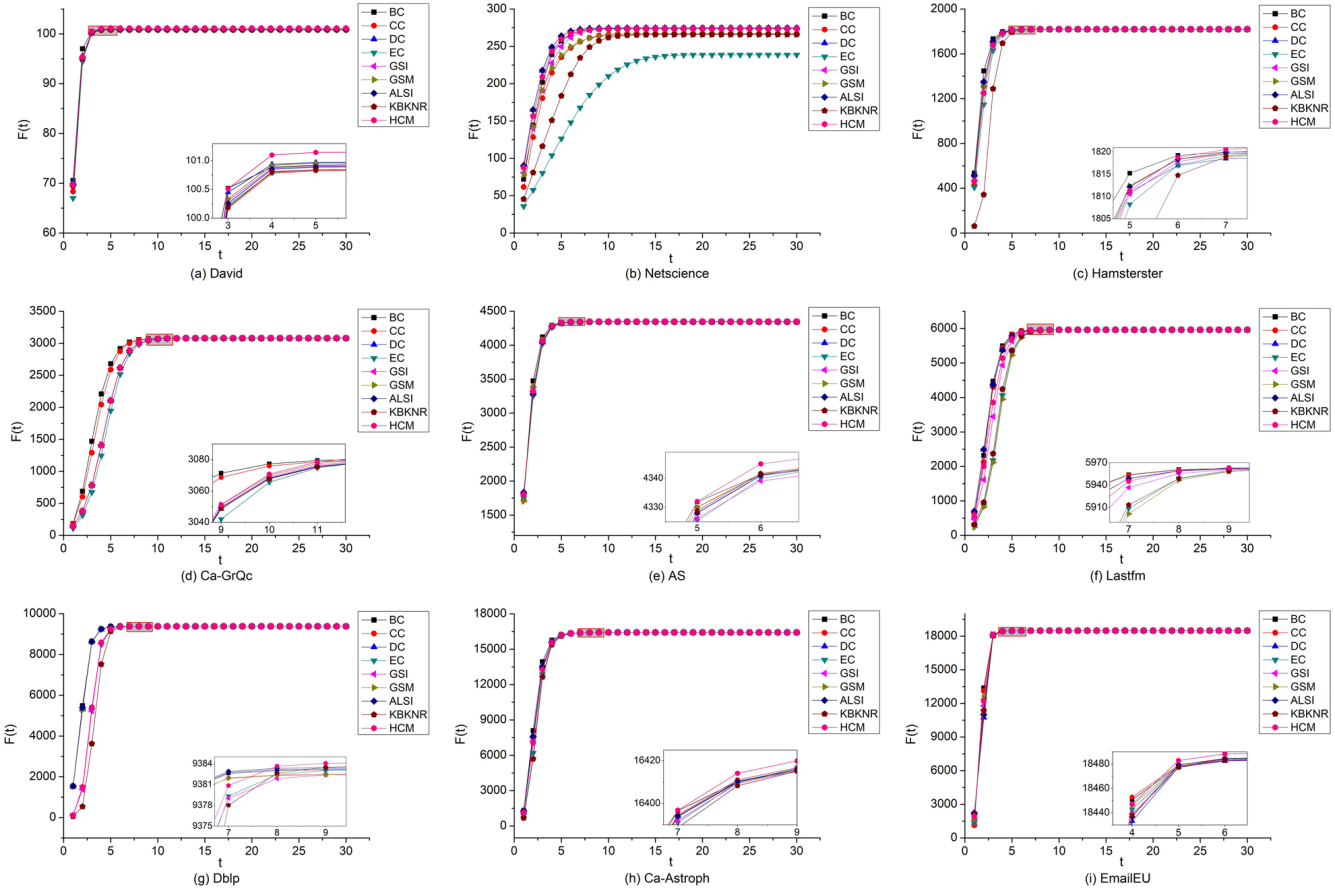
Infection values of the top ten nodes of the nine algorithms. The x-axis represents time t, and the y-axis represents the number of infective nodes at time t. As the infection values are close, the results in some networks are amplified.

In the previous experiment, the SIR model is used as the criteria to evaluate the output capacity and sorting results of important nodes excavated by different algorithms. Next, in the sequence of nodes sorted by various algorithms, the top ten nodes are selected as infective nodes. The SIR model is used to calculate their infection values and to measure the infection capacity of multiple nodes. For the SIR model, α is set to 0.5, β is set to 1, the infection time t is set to 30, and the number of iterations is set to 1000^55,56^. The infection results of each algorithm are shown in Fig. 6.

In Fig. 6, the infection values of ten infective nodes gradually increase with the increase in time t. When t≈5, the number of infective nodes reaches the maximum. At the beginning stage, because the infective nodes selected by BC pass the shortest path with the largest number and the infective nodes selected by DC have the most neighbors, the infective nodes selected by both BC and DC infect the most susceptible nodes. These two algorithms have the best effect. The infection values of nodes selected by the HCM are not maximum at the beginning stage, but when t > 9, they exceed those of other algorithms in David, Hamsterster, Ca-GrQc, AS, Lastfm, Dblp, Ca-Astroph, and EmailEU. Additionally, in these eight networks, the HCM can easily infect other nodes as well as more nodes. Netscience has a hierarchical organization structure, and the nodes identified by DC and BC have the strongest infection capacity, followed by HCM. The analysis of Fig. 6 shows that the HCM has the best overall performance in the evaluation of multi-node infection capacity.

To test the performance, we performed the experiment again by setting the value of α to 0.4 and β to 1, the results (Supplementary Tables S23–S31) suggest that the HCM method are still very competitive.

**Figure 7:**
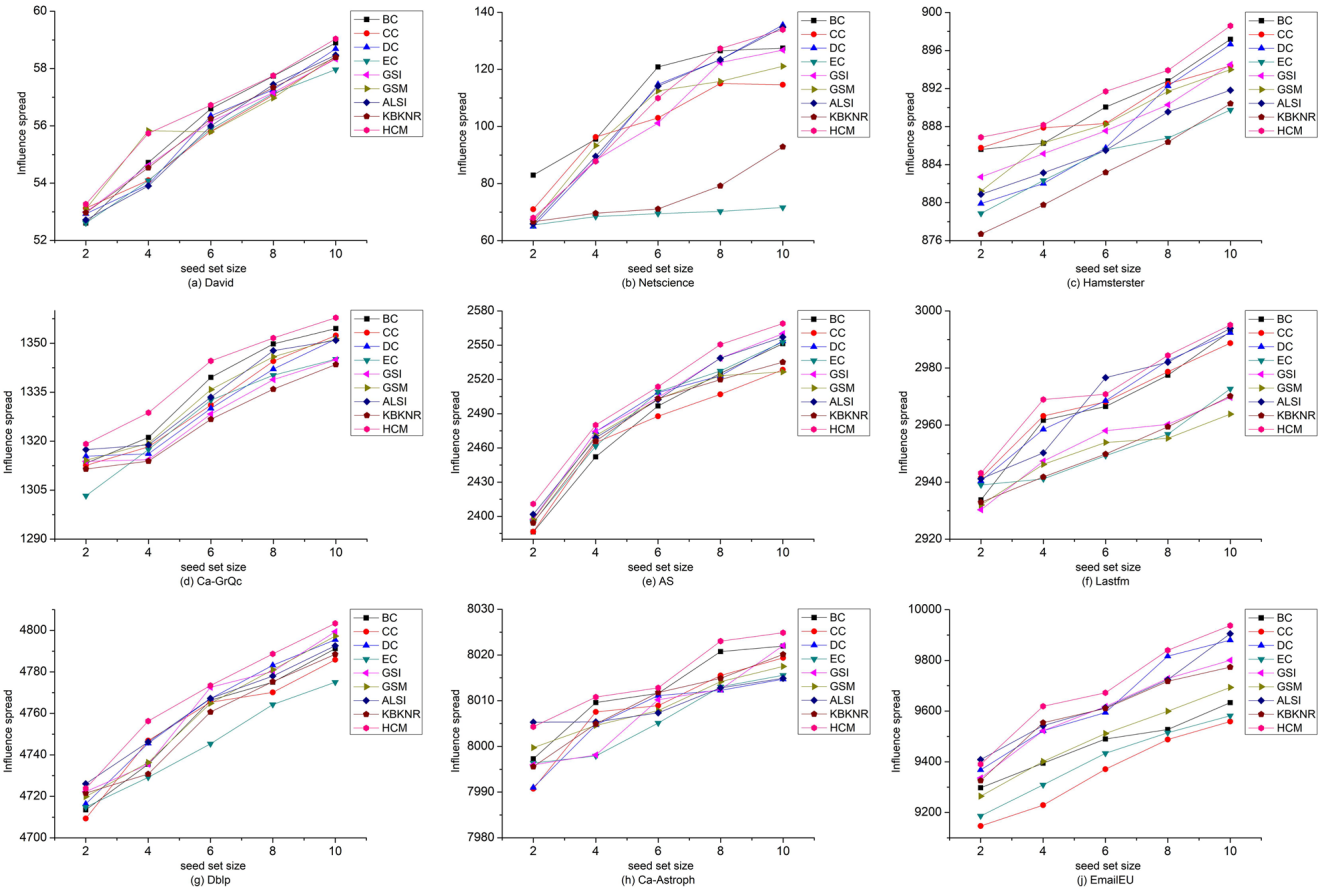
Comparison of the numbers of activated nodes by HCM and other algorithms on nine networks. The x-axis represents seed set size, and the y-axis represents the number of activated nodes.

In order to further demonstrate the effectiveness of the method, this study also conducted multi-node propagation experiments using the IC model. The experiments used the top 10 nodes identified by each method as the seed-set. Sequentially selecting 2, 4, 6, 8, and 10 seed nodes, the other nodes were activated with a propagation probability P set to 0.5, and the iteration was set to 1000 times. The average value was taken as the propagation value. The propagation results of each method are shown in Fig. 7.

From Fig. 7, it can be observed that as the number of seed nodes increases, the number of activated nodes gradually rises. Among the David, Hamsterster, Ca-GrQc, and AS networks, the HCM method outperforms other methods in selecting seed nodes for propagation. In the Dblp, Ca-Astroph, and EmailEU networks, when the number of seed nodes is 4, 6, 8, and 10, the HCM method outperforms all other algorithms. In the Lastfm network, when the number of seed nodes is 2, 4, 8, and 10, the HCM method shows a significant advantage. In the Netscience network, the HCM method performs worse than the BC, CC, and DC methods. Overall, the HCM method achieves good results in the David, Hamsterster, Ca-GrQc, AS, Lastfm, Dblp, Ca-Astroph, and EmailEU networks, and the experimental results in the IC model are consistent with the SIR model.

The source code is available at https://github.com/hhf602/HCM/blob/main/Code.

## Conclusion

In this paper, an important node excavating algorithm, the HCM, is proposed from the perspective of node output capacity. Inspired by degree centrality, eigenvector centrality, and closeness centrality, it considers degree value, eigenvector value, and distance between nodes when measuring the importance of nodes. Meanwhile, the network and degree densities of other nodes are introduced to reduce the influence of network structure characteristics on the algorithm accuracy. Finally, the output capacity of nodes is calculated by the HCM formula, which is used as an indicator to measure the importance of the nodes. Nine real networks are selected from real-world complex systems, and similarity experiments of output capability between nodes, comparison experiments of node importance sorting, and capability experiments of multi-node infection are carried out using the SIR model as the evaluation criterion. Furthermore, the top-2, top-4, top-6, top-8, and top-10 nodes of each algorithm were taken as seed nodes for multi-node concurrent propagation experiments in the IC model. Compared with eight algorithms for excavating important nodes, the experimental results show that the HCM outperforms other algorithms overall, verifying the accuracy and effectiveness of this algorithm.

The advantage of the HCM is that the output capacity of nodes is calculated through five attributes, namely the degree value, eigenvector value, distance, network density, and degree density. As the output capacity is the result of the combined influence of five attributes, it can avoid the accuracy of results being influenced by a too-large or too-small single attribute. At the same time, the influence of network structure characteristics on calculation results is reduced considering the network density and the degree density of other nodes, which makes the HCM a strong universal solution. As the HCM incorporates more attribute information, it improves accuracy but also increases time complexity. Future research will focus on how to reduce the time complexity while ensuring the accuracy.

### Supplementary Information

Supplementary Tables.

## Data Availability

All data generated or analysed during this study are included in this published article and its supplementary information files.
