# Supplementary material for: Excavating important nodes in complex networks based on the heat conduction model
Source: Sci Rep. 2024 Apr 2;14:7740. doi: 10.1038/s41598-024-58320-3 (PMC10987567; doi:10.1038/s41598-024-58320-3)
Supplement: Supplementary file 1 — Supplementary Tables. [file 41598_2024_58320_MOESM1_ESM.docx]

**Excavating Important Nodes in Complex Networks based**

**on the Heat Conduction Model**

All data generated or analysed during this study are included in this published article and its supplementary information files.

| (a) David | BC | CC | DC | EC | GSI | GSM | ALSI | KBKNR | HCM |
| --- | --- | --- | --- | --- | --- | --- | --- | --- | --- |
| 0.01 | 0.731981982 | 0.749517375 | 0.87467825 | 0.781370656 | 0.881435006 | 0.796010296 | 0.88497426 | 0.889317889 | 0.87532175 |
| 0.02 | 0.706241956 | 0.771074646 | 0.889478764 | 0.810006435 | 0.913288288 | 0.828507079 | 0.919401544 | 0.916184041 | 0.908783784 |
| 0.03 | 0.695624196 | 0.806467181 | 0.874195624 | 0.847972973 | 0.911357786 | 0.85971686 | 0.923584299 | 0.911357786 | 0.922297297 |
| 0.04 | 0.675032175 | 0.828024453 | 0.860521236 | 0.872425997 | 0.903957529 | 0.87998713 | 0.91489704 | 0.900740026 | 0.925514801 |
| 0.05 | 0.665057915 | 0.831885457 | 0.852477477 | 0.879504505 | 0.912001287 | 0.896074646 | 0.914253539 | 0.897522523 | 0.926158301 |
| 0.06 | 0.656692407 | 0.859877735 | 0.824163449 | 0.906531532 | 0.883043758 | 0.907979408 | 0.89494852 | 0.877252252 | 0.910714286 |
| 0.07 | 0.652027027 | 0.83976834 | 0.841055341 | 0.896396396 | 0.898970399 | 0.896235521 | 0.908944659 | 0.886100386 | 0.920849421 |
| 0.08 | 0.646396396 | 0.854407979 | 0.822876448 | 0.916505792 | 0.887226512 | 0.912483912 | 0.893983269 | 0.870173745 | 0.914253539 |
| 0.09 | 0.646235521 | 0.842342342 | 0.826093951 | 0.898005148 | 0.890604891 | 0.900096525 | 0.897361647 | 0.875482625 | 0.916988417 |
| 0.1 | 0.630630631 | 0.840572716 | 0.824163449 | 0.906853282 | 0.886583012 | 0.906048906 | 0.893339768 | 0.868886744 | 0.911036036 |

**Tab S1. Kendall τ values of the nine algorithms under ten infection probabilities in David.**

| (b) Netscience | BC | CC | DC | EC | GSI | GSM | ALSI | KBKNR | HCM |
| --- | --- | --- | --- | --- | --- | --- | --- | --- | --- |
| 0.01 | 0.39501054 | 0.168181374 | 0.810515 | 0.18854965 | 0.784073934 | 0.335539082 | 0.823763454 | 0.815247588 | 0.736929542 |
| 0.02 | 0.407588893 | 0.188256481 | 0.834108138 | 0.200848795 | 0.813837584 | 0.360221133 | 0.866761598 | 0.857212659 | 0.777707976 |
| 0.03 | 0.401320657 | 0.208582876 | 0.827602574 | 0.229300163 | 0.841409446 | 0.390599042 | 0.893439991 | 0.873350923 | 0.816043333 |
| 0.04 | 0.387276459 | 0.220449247 | 0.804051319 | 0.248635367 | 0.836844383 | 0.413787327 | 0.888874928 | 0.86172188 | 0.834219821 |
| 0.05 | 0.383758429 | 0.222040737 | 0.791403163 | 0.257863216 | 0.833382195 | 0.422177549 | 0.886082841 | 0.857031174 | 0.849325013 |
| 0.06 | 0.368290265 | 0.249123983 | 0.765771803 | 0.284820818 | 0.849003923 | 0.456883193 | 0.873420726 | 0.84986947 | 0.876575784 |
| 0.07 | 0.364074214 | 0.27240999 | 0.737320434 | 0.298306599 | 0.832698133 | 0.483826835 | 0.844550544 | 0.827253563 | 0.885440661 |
| 0.08 | 0.354595078 | 0.286845081 | 0.710251148 | 0.325724896 | 0.815722243 | 0.504627885 | 0.818988985 | 0.806159344 | 0.886501654 |
| 0.09 | 0.342240092 | 0.303485921 | 0.679831358 | 0.350211501 | 0.793245941 | 0.529728749 | 0.789909397 | 0.775460345 | 0.880051933 |
| 0.1 | 0.334687496 | 0.311206042 | 0.658164761 | 0.361952227 | 0.774162025 | 0.540883137 | 0.771020927 | 0.757437422 | 0.870126063 |

**Tab S2. Kendall τ values of the nine algorithms under ten infection probabilities in Netscience.**

| (c) Hamsterster | BC | CC | DC | EC | GSI | GSM | ALSI | KBKNR | HCM |
| --- | --- | --- | --- | --- | --- | --- | --- | --- | --- |
| 0.01 | 0.562522161 | 0.717159297 | 0.855622509 | 0.727062663 | 0.863537791 | 0.736007003 | 0.888557126 | 0.882930793 | 0.860901743 |
| 0.02 | 0.538838528 | 0.799951726 | 0.742729707 | 0.846507849 | 0.804141049 | 0.824116742 | 0.785249828 | 0.796134658 | 0.856928634 |
| 0.03 | 0.532260987 | 0.834363468 | 0.700827292 | 0.891172096 | 0.790625611 | 0.858955134 | 0.74565302 | 0.762151605 | 0.851768725 |
| 0.04 | 0.540010369 | 0.834080961 | 0.719699136 | 0.871417037 | 0.824334656 | 0.857083996 | 0.76649697 | 0.782975837 | 0.877907548 |
| 0.05 | 0.547076091 | 0.823073066 | 0.7414382 | 0.848480295 | 0.848254222 | 0.84404654 | 0.78801336 | 0.804685325 | 0.891422646 |
| 0.06 | 0.54953502 | 0.813045614 | 0.756027231 | 0.831690025 | 0.86506285 | 0.832411759 | 0.801594071 | 0.817979449 | 0.89737976 |
| 0.07 | 0.552495049 | 0.804285872 | 0.768570044 | 0.819696926 | 0.877401348 | 0.822892887 | 0.813212534 | 0.829581255 | 0.901510271 |
| 0.08 | 0.553471414 | 0.797815079 | 0.7772176 | 0.810975259 | 0.885700784 | 0.815658884 | 0.821298476 | 0.83746798 | 0.903404187 |
| 0.09 | 0.554567104 | 0.791040702 | 0.785840338 | 0.803020882 | 0.892619305 | 0.8086105 | 0.828967287 | 0.845221101 | 0.904572288 |
| 0.1 | 0.556865911 | 0.786454645 | 0.79308284 | 0.796103382 | 0.899022446 | 0.803641309 | 0.835279659 | 0.851977801 | 0.905611205 |

**Tab S3. Kendall τ values of the nine algorithms under ten infection probabilities in Hamsterster.**

| (d) Ca-GrQc | BC | CC | DC | EC | GSI | GSM | ALSI | KBKNR | HCM |
| --- | --- | --- | --- | --- | --- | --- | --- | --- | --- |
| 0.01 | 0.415276515 | 0.376040784 | 0.839518939 | 0.345834949 | 0.826303402 | 0.426885555 | 0.861132488 | 0.851307327 | 0.733042766 |
| 0.02 | 0.40417208 | 0.415380074 | 0.828968749 | 0.390660445 | 0.85249091 | 0.476048386 | 0.877043109 | 0.868093399 | 0.782603635 |
| 0.03 | 0.384739985 | 0.448800525 | 0.78688381 | 0.442423594 | 0.839757878 | 0.524706149 | 0.847974227 | 0.843718582 | 0.806810907 |
| 0.04 | 0.361605331 | 0.484960954 | 0.733943904 | 0.501244503 | 0.811798524 | 0.574305549 | 0.802751156 | 0.80002217 | 0.818515059 |
| 0.05 | 0.341043573 | 0.520884064 | 0.684162727 | 0.546494534 | 0.778520395 | 0.618401502 | 0.755697808 | 0.757129007 | 0.824026952 |
| 0.06 | 0.319386749 | 0.551655599 | 0.637372152 | 0.591917896 | 0.742096845 | 0.657268239 | 0.709709441 | 0.710844079 | 0.818017859 |
| 0.07 | 0.299630786 | 0.583622634 | 0.594337478 | 0.628095681 | 0.705423943 | 0.692285699 | 0.666036171 | 0.669620822 | 0.803663055 |
| 0.08 | 0.283114893 | 0.605551141 | 0.558802453 | 0.657209806 | 0.673944619 | 0.717224017 | 0.629091585 | 0.633338436 | 0.784859836 |
| 0.09 | 0.274638778 | 0.632633308 | 0.530397969 | 0.678955726 | 0.64887231 | 0.745005064 | 0.599110456 | 0.604616795 | 0.769349335 |
| 0.1 | 0.266379385 | 0.652144548 | 0.507957914 | 0.693341539 | 0.628580037 | 0.763027366 | 0.57542017 | 0.582216312 | 0.753985668 |

**Tab S4. Kendall τ values of the nine algorithms under ten infection probabilities in Ca-GrQc.**

| (e) AS | BC | CC | DC | EC | GSI | GSM | ALSI | KBKNR | HCM |
| --- | --- | --- | --- | --- | --- | --- | --- | --- | --- |
| 0.01 | 0.208194195 | 0.721670411 | 0.382063304 | 0.721662679 | 0.626082793 | 0.723780599 | 0.650356814 | 0.616863251 | 0.736370383 |
| 0.02 | 0.155827245 | 0.794549504 | 0.335629986 | 0.801170815 | 0.603952799 | 0.797795276 | 0.619474798 | 0.581416135 | 0.73893511 |
| 0.03 | 0.127187665 | 0.815189981 | 0.313550152 | 0.826125403 | 0.589249152 | 0.819163283 | 0.595762365 | 0.554980478 | 0.732919533 |
| 0.04 | 0.129415593 | 0.812873331 | 0.321623277 | 0.823761409 | 0.601067212 | 0.817036485 | 0.604496299 | 0.561174692 | 0.745063225 |
| 0.05 | 0.141835187 | 0.798132076 | 0.341178664 | 0.80665196 | 0.621067879 | 0.802433205 | 0.623883167 | 0.579843146 | 0.762075313 |
| 0.06 | 0.163248867 | 0.774058074 | 0.363015002 | 0.779389361 | 0.642101377 | 0.778391275 | 0.644869129 | 0.600172977 | 0.775158388 |
| 0.07 | 0.179191185 | 0.749829679 | 0.382623174 | 0.753984291 | 0.657393242 | 0.753954606 | 0.662555724 | 0.618326805 | 0.780886514 |
| 0.08 | 0.19399811 | 0.727804491 | 0.399600326 | 0.729985982 | 0.671711791 | 0.731663776 | 0.676985092 | 0.633898152 | 0.782096073 |
| 0.09 | 0.208362667 | 0.708097002 | 0.414524275 | 0.709166867 | 0.684025434 | 0.711628699 | 0.68977432 | 0.648000408 | 0.782839686 |
| 0.1 | 0.218976943 | 0.691646024 | 0.428131378 | 0.69261557 | 0.693262396 | 0.695016408 | 0.700192253 | 0.659140382 | 0.780960727 |

**Tab S5. Kendall τ values of the nine algorithms under ten infection probabilities in AS.**

| (f) Lastfm | BC | CC | DC | EC | GSI | GSM | ALSI | KBKNR | HCM |
| --- | --- | --- | --- | --- | --- | --- | --- | --- | --- |
| 0.01 | 0.567690953 | 0.557511997 | 0.8246246 | 0.443309163 | 0.848162296 | 0.768577009 | 0.866981435 | 0.864451536 | 0.813997176 |
| 0.02 | 0.528316718 | 0.62217847 | 0.764715504 | 0.516920613 | 0.818020387 | 0.748698298 | 0.833676499 | 0.829296185 | 0.851911237 |
| 0.03 | 0.469521877 | 0.666018328 | 0.674230517 | 0.600974931 | 0.740141257 | 0.685549537 | 0.750076982 | 0.744414959 | 0.823374208 |
| 0.04 | 0.411236174 | 0.68057536 | 0.590422871 | 0.672531897 | 0.664294517 | 0.618051848 | 0.665209246 | 0.658786183 | 0.76665794 |
| 0.05 | 0.375899949 | 0.696185565 | 0.541529239 | 0.699912103 | 0.618812373 | 0.576544151 | 0.615257039 | 0.608636824 | 0.731900436 |
| 0.06 | 0.365613763 | 0.705927304 | 0.534517921 | 0.685992672 | 0.613479957 | 0.574615618 | 0.608975413 | 0.60184895 | 0.729664286 |
| 0.07 | 0.374954386 | 0.714462734 | 0.552539988 | 0.66492737 | 0.631966185 | 0.594454686 | 0.629611827 | 0.622453257 | 0.749660895 |
| 0.08 | 0.386613164 | 0.711911569 | 0.575883045 | 0.645530887 | 0.656225898 | 0.619029862 | 0.653864657 | 0.646657978 | 0.770079648 |
| 0.09 | 0.399865397 | 0.709698579 | 0.59942962 | 0.630450641 | 0.680024995 | 0.642696641 | 0.679003723 | 0.671815386 | 0.790405451 |
| 0.1 | 0.410869712 | 0.702052412 | 0.6195921 | 0.614283636 | 0.700248523 | 0.662768684 | 0.699779269 | 0.692513537 | 0.804778719 |

**Tab S6. Kendall τ values of the nine algorithms under ten infection probabilities in Lastfm.**

| (g) Dblp | BC | CC | DC | EC | GSI | GSM | ALSI | KBKNR | HCM |
| --- | --- | --- | --- | --- | --- | --- | --- | --- | --- |
| 0.01 | 0.636789746 | 0.674845549 | 0.731580565 | 0.683754909 | 0.798767151 | 0.679572364 | 0.8051145 | 0.801639344 | 0.815553654 |
| 0.02 | 0.58798519 | 0.755578329 | 0.667653238 | 0.771181352 | 0.77764037 | 0.76220887 | 0.773310235 | 0.765801978 | 0.813917679 |
| 0.03 | 0.538798916 | 0.783897421 | 0.611024378 | 0.8140809 | 0.739004618 | 0.794137005 | 0.719028109 | 0.707507809 | 0.78088112 |
| 0.04 | 0.548432712 | 0.793687688 | 0.630778607 | 0.829075233 | 0.772649766 | 0.805283134 | 0.748983972 | 0.732561321 | 0.813411562 |
| 0.05 | 0.566282218 | 0.780639814 | 0.660516958 | 0.811788972 | 0.806329344 | 0.791434733 | 0.787020395 | 0.768514053 | 0.840169797 |
| 0.06 | 0.579693099 | 0.764253865 | 0.681903441 | 0.792821051 | 0.826720247 | 0.774511769 | 0.813443444 | 0.793684875 | 0.851923418 |
| 0.07 | 0.590372462 | 0.750333221 | 0.69882064 | 0.777141495 | 0.841230214 | 0.759940788 | 0.831947047 | 0.811710497 | 0.857456815 |
| 0.08 | 0.599755287 | 0.737456177 | 0.713297589 | 0.761318513 | 0.852451197 | 0.746200865 | 0.847700373 | 0.827451092 | 0.860686236 |
| 0.09 | 0.607629894 | 0.726978857 | 0.725019769 | 0.749509808 | 0.861234833 | 0.735171239 | 0.859572918 | 0.839506982 | 0.862368717 |
| 0.1 | 0.613467567 | 0.718082794 | 0.734248432 | 0.739589703 | 0.86754852 | 0.72593165 | 0.867711262 | 0.84786155 | 0.86279058 |

**Tab S7. Kendall τ values of the nine algorithms under ten infection probabilities in Dblp.**

| (h) Ca-Astroph | BC | CC | DC | EC | GSI | GSM | ALSI | KBKNR | HCM |
| --- | --- | --- | --- | --- | --- | --- | --- | --- | --- |
| 0.01 | 0.419072977 | 0.6696003 | 0.738996458 | 0.677089976 | 0.755117784 | 0.679646715 | 0.751783892 | 0.748742876 | 0.755117075 |
| 0.02 | 0.397802599 | 0.753115495 | 0.697027026 | 0.77045472 | 0.740685627 | 0.77090127 | 0.719072191 | 0.71817683 | 0.774023942 |
| 0.03 | 0.404091015 | 0.783259149 | 0.721363981 | 0.786944648 | 0.779147705 | 0.798661977 | 0.748046112 | 0.74877429 | 0.817959595 |
| 0.04 | 0.410244432 | 0.787261838 | 0.747660777 | 0.781588524 | 0.811083755 | 0.799687096 | 0.775740818 | 0.777510469 | 0.846492358 |
| 0.05 | 0.415830027 | 0.780152582 | 0.768329932 | 0.769346939 | 0.833127564 | 0.789290307 | 0.796483802 | 0.798947424 | 0.861301886 |
| 0.06 | 0.419287571 | 0.775406593 | 0.781826402 | 0.761826491 | 0.847510455 | 0.782572052 | 0.809994986 | 0.813056022 | 0.869940542 |
| 0.07 | 0.422779933 | 0.767769318 | 0.79361224 | 0.751607796 | 0.858494418 | 0.773152416 | 0.821276367 | 0.824747029 | 0.874718506 |
| 0.08 | 0.425038879 | 0.760962896 | 0.802091166 | 0.74316474 | 0.865960667 | 0.764825297 | 0.829287262 | 0.833263521 | 0.876544246 |
| 0.09 | 0.427589373 | 0.752123889 | 0.808266955 | 0.733946778 | 0.870279189 | 0.75517271 | 0.834668254 | 0.838863302 | 0.875680565 |
| 0.1 | 0.429033042 | 0.745439795 | 0.812586708 | 0.72607469 | 0.873128005 | 0.747373201 | 0.838286487 | 0.842861744 | 0.874296676 |

**Tab S8. Kendall τ values of the nine algorithms under ten infection probabilities in Ca-Astroph.**

| (i) EmailEU | BC | CC | DC | EC | GSI | GSM | ALSI | KBKNR | HCM |
| --- | --- | --- | --- | --- | --- | --- | --- | --- | --- |
| 0.01 | 0.149115235 | 0.270068218 | 0.215084419 | 0.277380706 | 0.291101654 | 0.265265329 | 0.306341886 | 0.305946215 | 0.299009533 |
| 0.02 | 0.143450571 | 0.38790454 | 0.214287618 | 0.396770683 | 0.380586652 | 0.376824461 | 0.393210886 | 0.382585356 | 0.397372045 |
| 0.03 | 0.154030026 | 0.46285556 | 0.237868199 | 0.478332352 | 0.45705418 | 0.454022376 | 0.453171106 | 0.429145002 | 0.473947194 |
| 0.04 | 0.171845069 | 0.489398641 | 0.272279988 | 0.508461715 | 0.508855629 | 0.485118413 | 0.502782708 | 0.474063384 | 0.523736716 |
| 0.05 | 0.182325975 | 0.482318549 | 0.290823703 | 0.504788014 | 0.518625794 | 0.478590455 | 0.513350608 | 0.483620247 | 0.532763137 |
| 0.06 | 0.190609614 | 0.465975761 | 0.308340404 | 0.488624708 | 0.522483524 | 0.465450691 | 0.519655039 | 0.492163187 | 0.533518406 |
| 0.07 | 0.195301474 | 0.446772456 | 0.317781018 | 0.468314204 | 0.513638913 | 0.445825142 | 0.513685581 | 0.489090504 | 0.523269841 |
| 0.08 | 0.199499211 | 0.428185074 | 0.327041774 | 0.449921704 | 0.504086191 | 0.425778256 | 0.50331033 | 0.480578331 | 0.513258252 |
| 0.09 | 0.203096293 | 0.4134145 | 0.332543158 | 0.434622304 | 0.493141968 | 0.4090452 | 0.49665256 | 0.476438639 | 0.502315532 |
| 0.1 | 0.206174715 | 0.39792678 | 0.338870571 | 0.41932194 | 0.486957138 | 0.396152547 | 0.485909882 | 0.467238957 | 0.492169611 |

**Tab S9. Kendall τ values of the nine algorithms under ten infection probabilities in EmailEU.**

| DataSets | \|Vertex\| | \|Edge\| | *Density*(G) | <*d*> | Max*d* | [Category](http://konect.cc/categories/) | Node meaning | Edge meaning |
| --- | --- | --- | --- | --- | --- | --- | --- | --- |
| Club-member | 25 | 90 | 0.3 | 4.750 | 21 | [Affiliation network](http://konect.cc/categories/Affiliation/) | Person, organization | Membership |
| South-africa | 6 | 8 | 0.5333333 | 2.364 | 4 | Affiliation network | Person, company | Leadership |
| Hiv | 40 | 41 | 0.052564 | 2.050 | 8 | [Co-authorship network](http://konect.cc/categories/Coauthorship/) | AIDS patient | Sexual contact |

**Tab S10. Statistical characteristics of three small actual networks.**

| (j) Club-member | BC | CC | DC | EC | GSI | GSM | AISI | KBKNR | HCM |
| --- | --- | --- | --- | --- | --- | --- | --- | --- | --- |
| 0.01 | 0.88 | 0.89 | 0.9 | 0.82 | 0.94 | 0.94 | 0.81 | 0.936666667 | 0.96 |
| 0.02 | 0.853333333 | 0.863333333 | 0.886666667 | 0.793333333 | 0.913333333 | 0.92 | 0.783333333 | 0.91 | 0.933333333 |
| 0.03 | 0.873333333 | 0.89 | 0.9 | 0.813333333 | 0.933333333 | 0.94 | 0.803333333 | 0.936666667 | 0.953333333 |
| 0.04 | 0.886666667 | 0.883333333 | 0.886666667 | 0.833333333 | 0.953333333 | 0.94 | 0.803333333 | 0.93 | 0.933333333 |
| 0.05 | 0.873333333 | 0.89 | 0.893333333 | 0.833333333 | 0.94 | 0.926666667 | 0.816666667 | 0.936666667 | 0.946666667 |
| 0.06 | 0.88 | 0.876666667 | 0.9 | 0.846666667 | 0.953333333 | 0.92 | 0.816666667 | 0.923333333 | 0.946666667 |
| 0.07 | 0.833333333 | 0.876666667 | 0.9 | 0.866666667 | 0.946666667 | 0.933333333 | 0.83 | 0.923333333 | 0.966666667 |
| 0.08 | 0.86 | 0.883333333 | 0.886666667 | 0.846666667 | 0.966666667 | 0.946666667 | 0.823333333 | 0.93 | 0.973333333 |
| 0.09 | 0.86 | 0.883333333 | 0.906666667 | 0.853333333 | 0.946666667 | 0.926666667 | 0.823333333 | 0.93 | 0.953333333 |
| 0.1 | 0.82 | 0.87 | 0.886666667 | 0.873333333 | 0.926666667 | 0.906666667 | 0.81 | 0.903333333 | 0.946666667 |

**Tab S11. Kendall τ values of the nine algorithms under ten infection probabilities in Club-member.**

| (k) South-africa | BC | CC | DC | EC | GSI | GSM | AISI | KBKNR | HCM |
| --- | --- | --- | --- | --- | --- | --- | --- | --- | --- |
| 0.01 | 0.666666667 | 0.8 | 0.533333333 | 0.866666667 | 0.866666667 | 0.866666667 | 0.866666667 | 0.8 | 0.866666667 |
| 0.02 | 0.8 | 0.8 | 0.4 | 0.733333333 | 0.733333333 | 0.733333333 | 0.733333333 | 0.8 | 0.733333333 |
| 0.03 | 0.8 | 0.933333333 | 0.4 | 0.6 | 0.866666667 | 0.866666667 | 0.866666667 | 0.933333333 | 0.866666667 |
| 0.04 | 0.666666667 | 0.8 | 0.533333333 | 0.866666667 | 0.866666667 | 0.866666667 | 0.866666667 | 0.8 | 0.866666667 |
| 0.05 | 0.666666667 | 0.933333333 | 0.533333333 | 0.733333333 | 1 | 1 | 1 | 0.933333333 | 1 |
| 0.06 | 0.666666667 | 0.933333333 | 0.533333333 | 0.733333333 | 1 | 1 | 1 | 0.933333333 | 1 |
| 0.07 | 0.666666667 | 0.866666667 | 0.533333333 | 0.8 | 0.933333333 | 0.933333333 | 0.933333333 | 0.866666667 | 0.933333333 |
| 0.08 | 0.666666667 | 0.933333333 | 0.533333333 | 0.733333333 | 1 | 1 | 1 | 0.933333333 | 1 |
| 0.09 | 0.666666667 | 0.933333333 | 0.533333333 | 0.733333333 | 1 | 1 | 1 | 0.933333333 | 1 |
| 0.1 | 0.666666667 | 0.933333333 | 0.533333333 | 0.733333333 | 1 | 1 | 1 | 0.933333333 | 1 |

**Tab S12. Kendall τ values of the nine algorithms under ten infection probabilities in South-africa.**

| (l) Hiv | BC | CC | DC | EC | GSI | GSM | AISI | KBKNR | HCM |
| --- | --- | --- | --- | --- | --- | --- | --- | --- | --- |
| 0.01 | 0.642307692 | 0.306410256 | 0.683333333 | 0.276923077 | 0.605128205 | 0.448717949 | 0.596153846 | 0.661538462 | 0.661538462 |
| 0.02 | 0.642307692 | 0.503846154 | 0.683333333 | 0.443589744 | 0.802564103 | 0.661538462 | 0.783333333 | 0.794871795 | 0.794871795 |
| 0.03 | 0.647435897 | 0.438461538 | 0.683333333 | 0.414102564 | 0.742307692 | 0.596153846 | 0.803846154 | 0.801282051 | 0.798717949 |
| 0.04 | 0.665384615 | 0.534615385 | 0.683333333 | 0.492307692 | 0.820512821 | 0.68974359 | 0.808974359 | 0.865384615 | 0.871794872 |
| 0.05 | 0.667948718 | 0.521794872 | 0.683333333 | 0.51025641 | 0.802564103 | 0.661538462 | 0.829487179 | 0.883333333 | 0.879487179 |
| 0.06 | 0.66025641 | 0.553846154 | 0.683333333 | 0.534615385 | 0.85 | 0.708974359 | 0.843589744 | 0.892307692 | 0.906410256 |
| 0.07 | 0.652564103 | 0.51025641 | 0.683333333 | 0.496153846 | 0.829487179 | 0.683333333 | 0.833333333 | 0.907692308 | 0.911538462 |
| 0.08 | 0.65 | 0.544871795 | 0.683333333 | 0.520512821 | 0.858974359 | 0.707692308 | 0.857692308 | 0.908974359 | 0.925641026 |
| 0.09 | 0.657692308 | 0.530769231 | 0.683333333 | 0.521794872 | 0.844871795 | 0.703846154 | 0.864102564 | 0.925641026 | 0.942307692 |
| 0.1 | 0.657692308 | 0.53974359 | 0.683333333 | 0.525641026 | 0.853846154 | 0.712820513 | 0.870512821 | 0.921794872 | 0.956410256 |

**Tab S13. Kendall τ values of the nine algorithms under ten infection probabilities in Hiv.**

| (a) David | BC | CC | DC | EC | GSI | GSM | ALSI | KBKNR | HCM |
| --- | --- | --- | --- | --- | --- | --- | --- | --- | --- |
| 1 | 70.552 | 68.341 | 70.223 | 67.014 | 69.428 | 69.683 | 69.603 | 69.468 | 69.675 |
| 2 | 97.03 | 95.298 | 95.499 | 94.47 | 95.097 | 95.304 | 95.282 | 95.055 | 95.354 |
| 3 | 100.525 | 100.175 | 100.449 | 100.221 | 100.304 | 100.34 | 100.258 | 100.195 | 100.516 |
| 4 | 100.889 | 100.783 | 100.94 | 100.8 | 100.875 | 100.921 | 100.855 | 100.812 | 101.101 |
| 5 | 100.924 | 100.826 | 100.97 | 100.844 | 100.905 | 100.959 | 100.89 | 100.843 | 101.147 |
| 6 | 100.928 | 100.828 | 100.971 | 100.85 | 100.908 | 100.96 | 100.895 | 100.848 | 101.149 |
| 7 | 100.929 | 100.828 | 100.971 | 100.853 | 100.908 | 100.96 | 100.895 | 100.848 | 101.149 |
| 8 | 100.929 | 100.828 | 100.971 | 100.853 | 100.908 | 100.96 | 100.895 | 100.848 | 101.149 |
| 9 | 100.929 | 100.828 | 100.971 | 100.853 | 100.908 | 100.96 | 100.895 | 100.848 | 101.149 |
| 10 | 100.929 | 100.828 | 100.971 | 100.853 | 100.908 | 100.96 | 100.895 | 100.848 | 101.149 |
| 11 | 100.929 | 100.828 | 100.971 | 100.853 | 100.908 | 100.96 | 100.895 | 100.848 | 101.149 |
| 12 | 100.929 | 100.828 | 100.971 | 100.853 | 100.908 | 100.96 | 100.895 | 100.848 | 101.149 |
| 13 | 100.929 | 100.828 | 100.971 | 100.853 | 100.908 | 100.96 | 100.895 | 100.848 | 101.149 |
| 14 | 100.929 | 100.828 | 100.971 | 100.853 | 100.908 | 100.96 | 100.895 | 100.848 | 101.149 |
| 15 | 100.929 | 100.828 | 100.971 | 100.853 | 100.908 | 100.96 | 100.895 | 100.848 | 101.149 |
| 16 | 100.929 | 100.828 | 100.971 | 100.853 | 100.908 | 100.96 | 100.895 | 100.848 | 101.149 |
| 17 | 100.929 | 100.828 | 100.971 | 100.853 | 100.908 | 100.96 | 100.895 | 100.848 | 101.149 |
| 18 | 100.929 | 100.828 | 100.971 | 100.853 | 100.908 | 100.96 | 100.895 | 100.848 | 101.149 |
| 19 | 100.929 | 100.828 | 100.971 | 100.853 | 100.908 | 100.96 | 100.895 | 100.848 | 101.149 |
| 20 | 100.929 | 100.828 | 100.971 | 100.853 | 100.908 | 100.96 | 100.895 | 100.848 | 101.149 |
| 21 | 100.929 | 100.828 | 100.971 | 100.853 | 100.908 | 100.96 | 100.895 | 100.848 | 101.149 |
| 22 | 100.929 | 100.828 | 100.971 | 100.853 | 100.908 | 100.96 | 100.895 | 100.848 | 101.149 |
| 23 | 100.929 | 100.828 | 100.971 | 100.853 | 100.908 | 100.96 | 100.895 | 100.848 | 101.149 |
| 24 | 100.929 | 100.828 | 100.971 | 100.853 | 100.908 | 100.96 | 100.895 | 100.848 | 101.149 |
| 25 | 100.929 | 100.828 | 100.971 | 100.853 | 100.908 | 100.96 | 100.895 | 100.848 | 101.149 |
| 26 | 100.929 | 100.828 | 100.971 | 100.853 | 100.908 | 100.96 | 100.895 | 100.848 | 101.149 |
| 27 | 100.929 | 100.828 | 100.971 | 100.853 | 100.908 | 100.96 | 100.895 | 100.848 | 101.149 |
| 28 | 100.929 | 100.828 | 100.971 | 100.853 | 100.908 | 100.96 | 100.895 | 100.848 | 101.149 |
| 29 | 100.929 | 100.828 | 100.971 | 100.853 | 100.908 | 100.96 | 100.895 | 100.848 | 101.149 |
| 30 | 100.929 | 100.828 | 100.971 | 100.853 | 100.908 | 100.96 | 100.895 | 100.848 | 101.149 |

**Tab S14. Infection values of the top ten nodes of the nine algorithms in David,** α **was set to 0.5,** *β* **was set to 1.**

| (b) Netscience | BC | CC | DC | EC | GSI | GSM | ALSI | KBKNR | HCM |
| --- | --- | --- | --- | --- | --- | --- | --- | --- | --- |
| 1 | 72.202 | 61.625 | 90.747 | 35.917 | 80.718 | 77.83 | 90.266 | 45.488 | 87.436 |
| 2 | 144.382 | 128.335 | 165.282 | 57.613 | 140.231 | 142.313 | 165.058 | 80.986 | 156.75 |
| 3 | 202.001 | 180.539 | 217.596 | 80.222 | 190.988 | 191.227 | 217.98 | 116.239 | 209.44 |
| 4 | 239.041 | 214.702 | 248.908 | 104.025 | 227.786 | 221.079 | 249.119 | 151.084 | 243.333 |
| 5 | 257.226 | 235.332 | 264.012 | 126.414 | 249.749 | 238.396 | 263.937 | 183.688 | 259.853 |
| 6 | 265.857 | 248.069 | 270.389 | 148.04 | 261.811 | 249.574 | 270.232 | 212.455 | 267.273 |
| 7 | 270.037 | 256.306 | 273.008 | 167.892 | 268.187 | 256.765 | 272.832 | 234.615 | 270.815 |
| 8 | 272.08 | 261.373 | 274.049 | 184.93 | 271.318 | 261.077 | 273.801 | 249.048 | 272.458 |
| 9 | 273.081 | 264.357 | 274.427 | 198.689 | 272.749 | 263.462 | 274.144 | 257.542 | 273.204 |
| 10 | 273.555 | 265.941 | 274.556 | 209.995 | 273.35 | 264.706 | 274.264 | 261.721 | 273.539 |
| 11 | 273.751 | 266.703 | 274.613 | 218.861 | 273.605 | 265.239 | 274.301 | 263.812 | 273.703 |
| 12 | 273.846 | 267.034 | 274.632 | 225.701 | 273.724 | 265.444 | 274.313 | 264.846 | 273.773 |
| 13 | 273.904 | 267.163 | 274.639 | 230.561 | 273.794 | 265.537 | 274.318 | 265.31 | 273.809 |
| 14 | 273.924 | 267.204 | 274.642 | 233.812 | 273.825 | 265.568 | 274.319 | 265.573 | 273.835 |
| 15 | 273.935 | 267.221 | 274.643 | 235.926 | 273.842 | 265.575 | 274.32 | 265.732 | 273.837 |
| 16 | 273.941 | 267.224 | 274.645 | 237.178 | 273.86 | 265.578 | 274.32 | 265.813 | 273.837 |
| 17 | 273.941 | 267.224 | 274.645 | 237.874 | 273.867 | 265.579 | 274.32 | 265.89 | 273.837 |
| 18 | 273.941 | 267.224 | 274.645 | 238.269 | 273.869 | 265.58 | 274.32 | 265.925 | 273.837 |
| 19 | 273.941 | 267.224 | 274.645 | 238.46 | 273.869 | 265.584 | 274.32 | 265.946 | 273.837 |
| 20 | 273.941 | 267.224 | 274.645 | 238.559 | 273.869 | 265.584 | 274.32 | 265.965 | 273.837 |
| 21 | 273.941 | 267.224 | 274.645 | 238.611 | 273.869 | 265.584 | 274.32 | 265.974 | 273.837 |
| 22 | 273.941 | 267.224 | 274.645 | 238.643 | 273.869 | 265.584 | 274.32 | 265.975 | 273.837 |
| 23 | 273.941 | 267.224 | 274.645 | 238.671 | 273.869 | 265.584 | 274.32 | 265.975 | 273.837 |
| 24 | 273.941 | 267.224 | 274.645 | 238.685 | 273.869 | 265.584 | 274.32 | 265.975 | 273.837 |
| 25 | 273.941 | 267.224 | 274.645 | 238.695 | 273.869 | 265.584 | 274.32 | 265.975 | 273.837 |
| 26 | 273.941 | 267.224 | 274.645 | 238.698 | 273.869 | 265.584 | 274.32 | 265.975 | 273.837 |
| 27 | 273.941 | 267.224 | 274.645 | 238.699 | 273.869 | 265.584 | 274.32 | 265.975 | 273.837 |
| 28 | 273.941 | 267.224 | 274.645 | 238.699 | 273.869 | 265.584 | 274.32 | 265.975 | 273.837 |
| 29 | 273.941 | 267.224 | 274.645 | 238.699 | 273.869 | 265.584 | 274.32 | 265.975 | 273.837 |
| 30 | 273.941 | 267.224 | 274.645 | 238.699 | 273.869 | 265.584 | 274.32 | 265.975 | 273.837 |

**Tab S15. Infection values of the top ten nodes of the nine algorithms in Netscience,** α **was set to 0.5,** *β* **was set to 1.**

| (c) Hamsterster | BC | CC | DC | EC | GSI | GSM | ALSI | KBKNR | HCM |
| --- | --- | --- | --- | --- | --- | --- | --- | --- | --- |
| 1 | 534.939 | 428.897 | 516.419 | 408.079 | 463.645 | 473.26 | 516.006 | 62.05 | 463.68 |
| 2 | 1447.57 | 1309.401 | 1352.614 | 1148.06 | 1248.265 | 1313.607 | 1351.005 | 342.715 | 1249.669 |
| 3 | 1732.776 | 1695.144 | 1710.356 | 1629.987 | 1677.117 | 1696.362 | 1710.714 | 1288.392 | 1677.76 |
| 4 | 1797.651 | 1786.643 | 1789.347 | 1775.654 | 1783.393 | 1788.102 | 1790.26 | 1694.694 | 1784.083 |
| 5 | 1815.232 | 1812.315 | 1811.03 | 1808.268 | 1810.546 | 1812.49 | 1812.179 | 1793.371 | 1811.366 |
| 6 | 1819.229 | 1818.432 | 1816.978 | 1817.056 | 1817.837 | 1818.368 | 1818.385 | 1814.767 | 1818.806 |
| 7 | 1819.966 | 1819.653 | 1818.487 | 1819.209 | 1819.62 | 1819.53 | 1819.953 | 1818.949 | 1820.597 |
| 8 | 1820.089 | 1819.84 | 1818.802 | 1819.633 | 1819.961 | 1819.691 | 1820.3 | 1819.602 | 1820.95 |
| 9 | 1820.114 | 1819.853 | 1818.856 | 1819.708 | 1820.019 | 1819.708 | 1820.363 | 1819.682 | 1821.019 |
| 10 | 1820.123 | 1819.857 | 1818.864 | 1819.715 | 1820.027 | 1819.71 | 1820.374 | 1819.699 | 1821.035 |
| 11 | 1820.124 | 1819.859 | 1818.865 | 1819.716 | 1820.028 | 1819.71 | 1820.375 | 1819.702 | 1821.036 |
| 12 | 1820.124 | 1819.859 | 1818.865 | 1819.716 | 1820.028 | 1819.71 | 1820.375 | 1819.705 | 1821.038 |
| 13 | 1820.124 | 1819.859 | 1818.865 | 1819.716 | 1820.028 | 1819.71 | 1820.375 | 1819.705 | 1821.038 |
| 14 | 1820.124 | 1819.859 | 1818.865 | 1819.716 | 1820.028 | 1819.71 | 1820.375 | 1819.705 | 1821.038 |
| 15 | 1820.124 | 1819.859 | 1818.865 | 1819.716 | 1820.028 | 1819.71 | 1820.375 | 1819.705 | 1821.038 |
| 16 | 1820.124 | 1819.859 | 1818.865 | 1819.716 | 1820.028 | 1819.71 | 1820.375 | 1819.705 | 1821.038 |
| 17 | 1820.124 | 1819.859 | 1818.865 | 1819.716 | 1820.028 | 1819.71 | 1820.375 | 1819.705 | 1821.038 |
| 18 | 1820.124 | 1819.859 | 1818.865 | 1819.716 | 1820.028 | 1819.71 | 1820.375 | 1819.705 | 1821.038 |
| 19 | 1820.124 | 1819.859 | 1818.865 | 1819.716 | 1820.028 | 1819.71 | 1820.375 | 1819.705 | 1821.038 |
| 20 | 1820.124 | 1819.859 | 1818.865 | 1819.716 | 1820.028 | 1819.71 | 1820.375 | 1819.705 | 1821.038 |
| 21 | 1820.124 | 1819.859 | 1818.865 | 1819.716 | 1820.028 | 1819.71 | 1820.375 | 1819.705 | 1821.038 |
| 22 | 1820.124 | 1819.859 | 1818.865 | 1819.716 | 1820.028 | 1819.71 | 1820.375 | 1819.705 | 1821.038 |
| 23 | 1820.124 | 1819.859 | 1818.865 | 1819.716 | 1820.028 | 1819.71 | 1820.375 | 1819.705 | 1821.038 |
| 24 | 1820.124 | 1819.859 | 1818.865 | 1819.716 | 1820.028 | 1819.71 | 1820.375 | 1819.705 | 1821.038 |
| 25 | 1820.124 | 1819.859 | 1818.865 | 1819.716 | 1820.028 | 1819.71 | 1820.375 | 1819.705 | 1821.038 |
| 26 | 1820.124 | 1819.859 | 1818.865 | 1819.716 | 1820.028 | 1819.71 | 1820.375 | 1819.705 | 1821.038 |
| 27 | 1820.124 | 1819.859 | 1818.865 | 1819.716 | 1820.028 | 1819.71 | 1820.375 | 1819.705 | 1821.038 |
| 28 | 1820.124 | 1819.859 | 1818.865 | 1819.716 | 1820.028 | 1819.71 | 1820.375 | 1819.705 | 1821.038 |
| 29 | 1820.124 | 1819.859 | 1818.865 | 1819.716 | 1820.028 | 1819.71 | 1820.375 | 1819.705 | 1821.038 |
| 30 | 1820.124 | 1819.859 | 1818.865 | 1819.716 | 1820.028 | 1819.71 | 1820.375 | 1819.705 | 1821.038 |

**Tab S16. Infection values of the top ten nodes of the nine algorithms in Hamsterster,** α **was set to 0.5,** *β* **was set to 1.**

| (d) Ca-GrQc | BC | CC | DC | EC | GSI | GSM | ALSI | KBKNR | HCM |
| --- | --- | --- | --- | --- | --- | --- | --- | --- | --- |
| 1 | 183.988 | 180.042 | 146.267 | 110.06 | 146.372 | 144.217 | 146.429 | 146.689 | 146.924 |
| 2 | 689.261 | 601.942 | 379.6 | 313.542 | 381.039 | 383.881 | 380.65 | 380.814 | 380.373 |
| 3 | 1468.788 | 1290.764 | 779.09 | 674.598 | 784.144 | 792.191 | 782.963 | 784.444 | 782.808 |
| 4 | 2209.978 | 2043.901 | 1401.382 | 1243.326 | 1412.345 | 1419.451 | 1407.517 | 1410.019 | 1407.44 |
| 5 | 2680.998 | 2588.088 | 2100.176 | 1945.771 | 2112.4 | 2114.932 | 2105.368 | 2108.228 | 2107.976 |
| 6 | 2917.982 | 2877.125 | 2613.502 | 2517.213 | 2620.699 | 2622.593 | 2617.838 | 2618.829 | 2619.448 |
| 7 | 3017.535 | 3000.432 | 2883.066 | 2839.869 | 2886.22 | 2889.349 | 2886.77 | 2886.166 | 2887.386 |
| 8 | 3056.457 | 3049.49 | 3000.469 | 2983.291 | 3002.224 | 3004.375 | 3002.344 | 3001.975 | 3003.568 |
| 9 | 3071.415 | 3068.688 | 3048.592 | 3042.101 | 3049.837 | 3051.217 | 3049.387 | 3048.797 | 3051.311 |
| 10 | 3077.314 | 3076.008 | 3067.994 | 3065.665 | 3068.933 | 3069.926 | 3068.211 | 3067.79 | 3070.768 |
| 11 | 3079.493 | 3078.711 | 3075.655 | 3074.845 | 3076.451 | 3077.499 | 3075.728 | 3075.384 | 3078.599 |
| 12 | 3080.283 | 3079.762 | 3078.576 | 3078.477 | 3079.422 | 3080.335 | 3078.61 | 3078.261 | 3081.634 |
| 13 | 3080.527 | 3080.129 | 3079.64 | 3079.885 | 3080.487 | 3081.381 | 3079.657 | 3079.28 | 3082.729 |
| 14 | 3080.618 | 3080.256 | 3080.012 | 3080.443 | 3080.851 | 3081.749 | 3080.031 | 3079.632 | 3083.131 |
| 15 | 3080.647 | 3080.295 | 3080.116 | 3080.648 | 3080.968 | 3081.834 | 3080.172 | 3079.764 | 3083.265 |
| 16 | 3080.661 | 3080.309 | 3080.16 | 3080.718 | 3081.024 | 3081.861 | 3080.231 | 3079.819 | 3083.316 |
| 17 | 3080.67 | 3080.314 | 3080.172 | 3080.743 | 3081.05 | 3081.868 | 3080.259 | 3079.835 | 3083.328 |
| 18 | 3080.672 | 3080.321 | 3080.175 | 3080.749 | 3081.051 | 3081.868 | 3080.271 | 3079.84 | 3083.337 |
| 19 | 3080.672 | 3080.323 | 3080.175 | 3080.753 | 3081.051 | 3081.868 | 3080.275 | 3079.841 | 3083.34 |
| 20 | 3080.672 | 3080.324 | 3080.175 | 3080.756 | 3081.051 | 3081.868 | 3080.275 | 3079.841 | 3083.34 |
| 21 | 3080.672 | 3080.324 | 3080.175 | 3080.758 | 3081.051 | 3081.868 | 3080.275 | 3079.841 | 3083.34 |
| 22 | 3080.672 | 3080.324 | 3080.175 | 3080.758 | 3081.051 | 3081.868 | 3080.275 | 3079.841 | 3083.34 |
| 23 | 3080.672 | 3080.324 | 3080.175 | 3080.758 | 3081.051 | 3081.868 | 3080.275 | 3079.841 | 3083.34 |
| 24 | 3080.672 | 3080.324 | 3080.175 | 3080.758 | 3081.051 | 3081.868 | 3080.275 | 3079.841 | 3083.34 |
| 25 | 3080.672 | 3080.324 | 3080.175 | 3080.758 | 3081.051 | 3081.868 | 3080.275 | 3079.841 | 3083.34 |
| 26 | 3080.672 | 3080.324 | 3080.175 | 3080.758 | 3081.051 | 3081.868 | 3080.275 | 3079.841 | 3083.34 |
| 27 | 3080.672 | 3080.324 | 3080.175 | 3080.758 | 3081.051 | 3081.868 | 3080.275 | 3079.841 | 3083.34 |
| 28 | 3080.672 | 3080.324 | 3080.175 | 3080.758 | 3081.051 | 3081.868 | 3080.275 | 3079.841 | 3083.34 |
| 29 | 3080.672 | 3080.324 | 3080.175 | 3080.758 | 3081.051 | 3081.868 | 3080.275 | 3079.841 | 3083.34 |
| 30 | 3080.672 | 3080.324 | 3080.175 | 3080.758 | 3081.051 | 3081.868 | 3080.275 | 3079.841 | 3083.34 |

**Tab S17. Infection values of the top ten nodes of the nine algorithms in Ca-GrQc,** α **was set to 0.5,** *β* **was set to 1.**

| (e) AS | BC | CC | DC | EC | GSI | GSM | ALSI | KBKNR | HCM |
| --- | --- | --- | --- | --- | --- | --- | --- | --- | --- |
| 1 | 1767.253 | 1716.925 | 1834.578 | 1757.369 | 1804.555 | 1703.1 | 1833.291 | 1805.898 | 1805.634 |
| 2 | 3473.673 | 3381.721 | 3286.096 | 3251.573 | 3317.652 | 3384.581 | 3287.258 | 3321.315 | 3319.281 |
| 3 | 4121.909 | 4082.181 | 4043.176 | 4021.293 | 4052.264 | 4079.126 | 4044.025 | 4057.532 | 4058.776 |
| 4 | 4293.973 | 4284.36 | 4275.183 | 4267.561 | 4275.865 | 4283.677 | 4274.82 | 4278.753 | 4281.992 |
| 5 | 4331.858 | 4329.959 | 4328.006 | 4325.311 | 4326.156 | 4330.033 | 4328.087 | 4328.759 | 4332.026 |
| 6 | 4341.362 | 4341.248 | 4340.886 | 4339.932 | 4339.083 | 4341.57 | 4341.061 | 4341.636 | 4344.974 |
| 7 | 4343.671 | 4343.728 | 4343.702 | 4343.5 | 4341.908 | 4344.034 | 4344.088 | 4344.45 | 4347.714 |
| 8 | 4344.363 | 4344.584 | 4344.613 | 4344.525 | 4342.84 | 4344.898 | 4345.001 | 4345.271 | 4348.429 |
| 9 | 4344.533 | 4344.772 | 4344.86 | 4344.748 | 4343.059 | 4345.098 | 4345.209 | 4345.426 | 4348.6 |
| 10 | 4344.553 | 4344.814 | 4344.945 | 4344.843 | 4343.137 | 4345.143 | 4345.258 | 4345.486 | 4348.649 |
| 11 | 4344.562 | 4344.819 | 4344.967 | 4344.858 | 4343.155 | 4345.158 | 4345.266 | 4345.498 | 4348.661 |
| 12 | 4344.563 | 4344.819 | 4344.967 | 4344.864 | 4343.158 | 4345.16 | 4345.266 | 4345.498 | 4348.661 |
| 13 | 4344.563 | 4344.819 | 4344.967 | 4344.864 | 4343.159 | 4345.164 | 4345.266 | 4345.498 | 4348.661 |
| 14 | 4344.563 | 4344.819 | 4344.967 | 4344.864 | 4343.16 | 4345.164 | 4345.266 | 4345.498 | 4348.661 |
| 15 | 4344.563 | 4344.819 | 4344.967 | 4344.864 | 4343.16 | 4345.164 | 4345.266 | 4345.498 | 4348.661 |
| 16 | 4344.563 | 4344.819 | 4344.967 | 4344.864 | 4343.16 | 4345.164 | 4345.266 | 4345.498 | 4348.661 |
| 17 | 4344.563 | 4344.819 | 4344.967 | 4344.864 | 4343.16 | 4345.164 | 4345.266 | 4345.498 | 4348.661 |
| 18 | 4344.563 | 4344.819 | 4344.967 | 4344.864 | 4343.16 | 4345.164 | 4345.266 | 4345.498 | 4348.661 |
| 19 | 4344.563 | 4344.819 | 4344.967 | 4344.864 | 4343.16 | 4345.164 | 4345.266 | 4345.498 | 4348.661 |
| 20 | 4344.563 | 4344.819 | 4344.967 | 4344.864 | 4343.16 | 4345.164 | 4345.266 | 4345.498 | 4348.661 |
| 21 | 4344.563 | 4344.819 | 4344.967 | 4344.864 | 4343.16 | 4345.164 | 4345.266 | 4345.498 | 4348.661 |
| 22 | 4344.563 | 4344.819 | 4344.967 | 4344.864 | 4343.16 | 4345.164 | 4345.266 | 4345.498 | 4348.661 |
| 23 | 4344.563 | 4344.819 | 4344.967 | 4344.864 | 4343.16 | 4345.164 | 4345.266 | 4345.498 | 4348.661 |
| 24 | 4344.563 | 4344.819 | 4344.967 | 4344.864 | 4343.16 | 4345.164 | 4345.266 | 4345.498 | 4348.661 |
| 25 | 4344.563 | 4344.819 | 4344.967 | 4344.864 | 4343.16 | 4345.164 | 4345.266 | 4345.498 | 4348.661 |
| 26 | 4344.563 | 4344.819 | 4344.967 | 4344.864 | 4343.16 | 4345.164 | 4345.266 | 4345.498 | 4348.661 |
| 27 | 4344.563 | 4344.819 | 4344.967 | 4344.864 | 4343.16 | 4345.164 | 4345.266 | 4345.498 | 4348.661 |
| 28 | 4344.563 | 4344.819 | 4344.967 | 4344.864 | 4343.16 | 4345.164 | 4345.266 | 4345.498 | 4348.661 |
| 29 | 4344.563 | 4344.819 | 4344.967 | 4344.864 | 4343.16 | 4345.164 | 4345.266 | 4345.498 | 4348.661 |
| 30 | 4344.563 | 4344.819 | 4344.967 | 4344.864 | 4343.16 | 4345.164 | 4345.266 | 4345.498 | 4348.661 |

**Tab S18. Infection values of the top ten nodes of the nine algorithms in AS,** α **was set to 0.5,** *β* **was set to 1.**

| (f) Lastfm | BC | CC | DC | EC | GSI | GSM | ALSI | KBKNR | HCM |
| --- | --- | --- | --- | --- | --- | --- | --- | --- | --- |
| 1 | 556.757 | 503.783 | 701.996 | 252.262 | 468.341 | 238.119 | 702.028 | 314.331 | 593.009 |
| 2 | 2316.249 | 2122.839 | 2491.341 | 867.272 | 1612.303 | 821.452 | 2490.912 | 960.77 | 2006.258 |
| 3 | 4466.864 | 4301.36 | 4375.54 | 2177.076 | 3443.091 | 2110.485 | 4378.114 | 2372.676 | 3854.042 |
| 4 | 5495.308 | 5444.18 | 5376.274 | 4063.657 | 4935.798 | 3949.675 | 5380.554 | 4244.035 | 5139.198 |
| 5 | 5834.971 | 5825.569 | 5780.835 | 5289.109 | 5630.586 | 5223.519 | 5782.857 | 5365.493 | 5707.018 |
| 6 | 5929.233 | 5927.468 | 5911.03 | 5761.827 | 5867.218 | 5738.165 | 5911.861 | 5785.083 | 5892 |
| 7 | 5954.191 | 5953.575 | 5948.987 | 5908.509 | 5936.725 | 5901.217 | 5948.851 | 5913.412 | 5945.321 |
| 8 | 5960.927 | 5960.376 | 5959.413 | 5949.288 | 5955.777 | 5946.542 | 5958.99 | 5948.88 | 5959.625 |
| 9 | 5962.567 | 5962.127 | 5962.352 | 5959.912 | 5960.885 | 5958.429 | 5961.646 | 5958.596 | 5963.365 |
| 10 | 5962.976 | 5962.585 | 5963.108 | 5962.627 | 5962.123 | 5961.561 | 5962.268 | 5961.056 | 5964.211 |
| 11 | 5963.084 | 5962.706 | 5963.33 | 5963.325 | 5962.453 | 5962.361 | 5962.434 | 5961.627 | 5964.417 |
| 12 | 5963.134 | 5962.758 | 5963.382 | 5963.523 | 5962.517 | 5962.567 | 5962.474 | 5961.784 | 5964.469 |
| 13 | 5963.141 | 5962.789 | 5963.393 | 5963.573 | 5962.533 | 5962.608 | 5962.483 | 5961.837 | 5964.489 |
| 14 | 5963.143 | 5962.8 | 5963.394 | 5963.599 | 5962.534 | 5962.626 | 5962.487 | 5961.858 | 5964.495 |
| 15 | 5963.143 | 5962.802 | 5963.396 | 5963.611 | 5962.534 | 5962.643 | 5962.487 | 5961.864 | 5964.495 |
| 16 | 5963.143 | 5962.802 | 5963.396 | 5963.614 | 5962.534 | 5962.658 | 5962.487 | 5961.864 | 5964.495 |
| 17 | 5963.143 | 5962.802 | 5963.396 | 5963.615 | 5962.534 | 5962.667 | 5962.487 | 5961.864 | 5964.495 |
| 18 | 5963.143 | 5962.802 | 5963.396 | 5963.615 | 5962.534 | 5962.668 | 5962.487 | 5961.864 | 5964.495 |
| 19 | 5963.143 | 5962.802 | 5963.396 | 5963.615 | 5962.534 | 5962.668 | 5962.487 | 5961.864 | 5964.495 |
| 20 | 5963.143 | 5962.802 | 5963.396 | 5963.615 | 5962.534 | 5962.668 | 5962.487 | 5961.864 | 5964.495 |
| 21 | 5963.143 | 5962.802 | 5963.396 | 5963.615 | 5962.534 | 5962.668 | 5962.487 | 5961.864 | 5964.495 |
| 22 | 5963.143 | 5962.802 | 5963.396 | 5963.615 | 5962.534 | 5962.668 | 5962.487 | 5961.864 | 5964.495 |
| 23 | 5963.143 | 5962.802 | 5963.396 | 5963.615 | 5962.534 | 5962.668 | 5962.487 | 5961.864 | 5964.495 |
| 24 | 5963.143 | 5962.802 | 5963.396 | 5963.615 | 5962.534 | 5962.668 | 5962.487 | 5961.864 | 5964.495 |
| 25 | 5963.143 | 5962.802 | 5963.396 | 5963.615 | 5962.534 | 5962.668 | 5962.487 | 5961.864 | 5964.495 |
| 26 | 5963.143 | 5962.802 | 5963.396 | 5963.615 | 5962.534 | 5962.668 | 5962.487 | 5961.864 | 5964.495 |
| 27 | 5963.143 | 5962.802 | 5963.396 | 5963.615 | 5962.534 | 5962.668 | 5962.487 | 5961.864 | 5964.495 |
| 28 | 5963.143 | 5962.802 | 5963.396 | 5963.615 | 5962.534 | 5962.668 | 5962.487 | 5961.864 | 5964.495 |
| 29 | 5963.143 | 5962.802 | 5963.396 | 5963.615 | 5962.534 | 5962.668 | 5962.487 | 5961.864 | 5964.495 |
| 30 | 5963.143 | 5962.802 | 5963.396 | 5963.615 | 5962.534 | 5962.668 | 5962.487 | 5961.864 | 5964.495 |

**Tab S19. Infection values of the top ten nodes of the nine algorithms in Lastfm,** α **was set to 0.5,** *β* **was set to 1.**

| (g) Dblp | BC | CC | DC | EC | GSI | GSM | ALSI | KBKNR | HCM |
| --- | --- | --- | --- | --- | --- | --- | --- | --- | --- |
| 1 | 1531.461 | 1533.729 | 1530.036 | 111.007 | 111.28 | 1525.194 | 1551.925 | 60.219 | 116.93 |
| 2 | 5486.725 | 5338.94 | 5310.665 | 1470.257 | 1376.752 | 5285.343 | 5393.646 | 538.914 | 1504.067 |
| 3 | 8638.408 | 8645.648 | 8621.876 | 5261.354 | 5226.19 | 8632.176 | 8636.572 | 3626.039 | 5405.781 |
| 4 | 9252.79 | 9246.375 | 9242.877 | 8488.247 | 8529.625 | 9241.857 | 9247.023 | 7521.965 | 8594.625 |
| 5 | 9365.683 | 9364.696 | 9365.369 | 9236.721 | 9241.412 | 9364.279 | 9365.701 | 9134.157 | 9253.351 |
| 6 | 9379.868 | 9378.95 | 9379.81 | 9359.147 | 9361.424 | 9379.13 | 9380.03 | 9343.257 | 9363.693 |
| 7 | 9382.606 | 9381.943 | 9382.745 | 9379.348 | 9379.029 | 9381.918 | 9382.894 | 9378.032 | 9380.874 |
| 8 | 9383.027 | 9382.38 | 9383.187 | 9382.447 | 9381.884 | 9382.315 | 9383.38 | 9382.675 | 9383.675 |
| 9 | 9383.144 | 9382.504 | 9383.275 | 9383.057 | 9382.397 | 9382.411 | 9383.459 | 9383.519 | 9384.089 |
| 10 | 9383.147 | 9382.537 | 9383.281 | 9383.18 | 9382.489 | 9382.417 | 9383.479 | 9383.707 | 9384.149 |
| 11 | 9383.15 | 9382.537 | 9383.283 | 9383.197 | 9382.516 | 9382.417 | 9383.481 | 9383.736 | 9384.168 |
| 12 | 9383.15 | 9382.537 | 9383.283 | 9383.2 | 9382.52 | 9382.417 | 9383.481 | 9383.737 | 9384.169 |
| 13 | 9383.15 | 9382.537 | 9383.283 | 9383.2 | 9382.52 | 9382.417 | 9383.481 | 9383.74 | 9384.169 |
| 14 | 9383.15 | 9382.537 | 9383.283 | 9383.2 | 9382.52 | 9382.417 | 9383.481 | 9383.74 | 9384.169 |
| 15 | 9383.15 | 9382.537 | 9383.283 | 9383.2 | 9382.52 | 9382.417 | 9383.481 | 9383.74 | 9384.169 |
| 16 | 9383.15 | 9382.537 | 9383.283 | 9383.2 | 9382.52 | 9382.417 | 9383.481 | 9383.74 | 9384.169 |
| 17 | 9383.15 | 9382.537 | 9383.283 | 9383.2 | 9382.52 | 9382.417 | 9383.481 | 9383.74 | 9384.169 |
| 18 | 9383.15 | 9382.537 | 9383.283 | 9383.2 | 9382.52 | 9382.417 | 9383.481 | 9383.74 | 9384.169 |
| 19 | 9383.15 | 9382.537 | 9383.283 | 9383.2 | 9382.52 | 9382.417 | 9383.481 | 9383.74 | 9384.169 |
| 20 | 9383.15 | 9382.537 | 9383.283 | 9383.2 | 9382.52 | 9382.417 | 9383.481 | 9383.74 | 9384.169 |
| 21 | 9383.15 | 9382.537 | 9383.283 | 9383.2 | 9382.52 | 9382.417 | 9383.481 | 9383.74 | 9384.169 |
| 22 | 9383.15 | 9382.537 | 9383.283 | 9383.2 | 9382.52 | 9382.417 | 9383.481 | 9383.74 | 9384.169 |
| 23 | 9383.15 | 9382.537 | 9383.283 | 9383.2 | 9382.52 | 9382.417 | 9383.481 | 9383.74 | 9384.169 |
| 24 | 9383.15 | 9382.537 | 9383.283 | 9383.2 | 9382.52 | 9382.417 | 9383.481 | 9383.74 | 9384.169 |
| 25 | 9383.15 | 9382.537 | 9383.283 | 9383.2 | 9382.52 | 9382.417 | 9383.481 | 9383.74 | 9384.169 |
| 26 | 9383.15 | 9382.537 | 9383.283 | 9383.2 | 9382.52 | 9382.417 | 9383.481 | 9383.74 | 9384.169 |
| 27 | 9383.15 | 9382.537 | 9383.283 | 9383.2 | 9382.52 | 9382.417 | 9383.481 | 9383.74 | 9384.169 |
| 28 | 9383.15 | 9382.537 | 9383.283 | 9383.2 | 9382.52 | 9382.417 | 9383.481 | 9383.74 | 9384.169 |
| 29 | 9383.15 | 9382.537 | 9383.283 | 9383.2 | 9382.52 | 9382.417 | 9383.481 | 9383.74 | 9384.169 |
| 30 | 9383.15 | 9382.537 | 9383.283 | 9383.2 | 9382.52 | 9382.417 | 9383.481 | 9383.74 | 9384.169 |

**Tab S20. Infection values of the top ten nodes of the nine algorithms in Dblp,** α **was set to 0.5,** *β* **was set to 1.**

| (h) Ca-Astroph | BC | CC | DC | EC | GSI | GSM | ALSI | KBKNR | HCM |
| --- | --- | --- | --- | --- | --- | --- | --- | --- | --- |
| 1 | 1288.292 | 1235.966 | 1287.595 | 880.209 | 1181.342 | 1286.282 | 1287.162 | 692.347 | 1132.082 |
| 2 | 8093.847 | 7506.101 | 7612.655 | 6195.907 | 7205.275 | 7626.529 | 7614.876 | 5701.973 | 7072.648 |
| 3 | 13928.506 | 13477.644 | 13536.281 | 12863.4 | 13343.092 | 13540.533 | 13535.251 | 12649.011 | 13272.208 |
| 4 | 15768.6 | 15621.616 | 15635.354 | 15434.019 | 15571.69 | 15634.703 | 15634.34 | 15384.862 | 15556.897 |
| 5 | 16233.502 | 16192.908 | 16195.567 | 16148.883 | 16179.756 | 16194.772 | 16195.851 | 16135.045 | 16182.008 |
| 6 | 16356.827 | 16346.052 | 16346.214 | 16335.011 | 16341.428 | 16345.522 | 16346.494 | 16330.494 | 16345.512 |
| 7 | 16396.584 | 16394.644 | 16393.881 | 16391.084 | 16392.082 | 16393.766 | 16394.132 | 16388.719 | 16396.889 |
| 8 | 16410.308 | 16411.064 | 16410.112 | 16409.889 | 16409.372 | 16409.918 | 16410.41 | 16408.296 | 16414.134 |
| 9 | 16415.147 | 16416.747 | 16415.897 | 16416.47 | 16415.384 | 16415.574 | 16415.98 | 16415.105 | 16419.984 |
| 10 | 16416.694 | 16418.63 | 16417.844 | 16418.72 | 16417.374 | 16417.491 | 16417.853 | 16417.38 | 16421.986 |
| 11 | 16417.148 | 16419.224 | 16418.498 | 16419.455 | 16417.969 | 16418.049 | 16418.401 | 16418.124 | 16422.537 |
| 12 | 16417.251 | 16419.386 | 16418.653 | 16419.667 | 16418.126 | 16418.19 | 16418.56 | 16418.309 | 16422.681 |
| 13 | 16417.271 | 16419.427 | 16418.678 | 16419.71 | 16418.154 | 16418.221 | 16418.595 | 16418.352 | 16422.713 |
| 14 | 16417.274 | 16419.432 | 16418.682 | 16419.714 | 16418.16 | 16418.222 | 16418.608 | 16418.359 | 16422.721 |
| 15 | 16417.275 | 16419.433 | 16418.682 | 16419.714 | 16418.161 | 16418.222 | 16418.61 | 16418.361 | 16422.724 |
| 16 | 16417.275 | 16419.433 | 16418.682 | 16419.714 | 16418.161 | 16418.222 | 16418.61 | 16418.361 | 16422.724 |
| 17 | 16417.275 | 16419.433 | 16418.682 | 16419.714 | 16418.161 | 16418.222 | 16418.61 | 16418.361 | 16422.724 |
| 18 | 16417.275 | 16419.433 | 16418.682 | 16419.714 | 16418.161 | 16418.222 | 16418.61 | 16418.361 | 16422.724 |
| 19 | 16417.275 | 16419.433 | 16418.682 | 16419.714 | 16418.161 | 16418.222 | 16418.61 | 16418.361 | 16422.724 |
| 20 | 16417.275 | 16419.433 | 16418.682 | 16419.714 | 16418.161 | 16418.222 | 16418.61 | 16418.361 | 16422.724 |
| 21 | 16417.275 | 16419.433 | 16418.682 | 16419.714 | 16418.161 | 16418.222 | 16418.61 | 16418.361 | 16422.724 |
| 22 | 16417.275 | 16419.433 | 16418.682 | 16419.714 | 16418.161 | 16418.222 | 16418.61 | 16418.361 | 16422.724 |
| 23 | 16417.275 | 16419.433 | 16418.682 | 16419.714 | 16418.161 | 16418.222 | 16418.61 | 16418.361 | 16422.724 |
| 24 | 16417.275 | 16419.433 | 16418.682 | 16419.714 | 16418.161 | 16418.222 | 16418.61 | 16418.361 | 16422.724 |
| 25 | 16417.275 | 16419.433 | 16418.682 | 16419.714 | 16418.161 | 16418.222 | 16418.61 | 16418.361 | 16422.724 |
| 26 | 16417.275 | 16419.433 | 16418.682 | 16419.714 | 16418.161 | 16418.222 | 16418.61 | 16418.361 | 16422.724 |
| 27 | 16417.275 | 16419.433 | 16418.682 | 16419.714 | 16418.161 | 16418.222 | 16418.61 | 16418.361 | 16422.724 |
| 28 | 16417.275 | 16419.433 | 16418.682 | 16419.714 | 16418.161 | 16418.222 | 16418.61 | 16418.361 | 16422.724 |
| 29 | 16417.275 | 16419.433 | 16418.682 | 16419.714 | 16418.161 | 16418.222 | 16418.61 | 16418.361 | 16422.724 |
| 30 | 16417.275 | 16419.433 | 16418.682 | 16419.714 | 16418.161 | 16418.222 | 16418.61 | 16418.361 | 16422.724 |

**Tab S21. Infection values of the top ten nodes of the nine algorithms in Ca-Astroph,** α **was set to 0.5,** *β* **was set to 1.**

| (i) EmailEU | BC | CC | DC | EC | GSI | GSM | ALSI | KBKNR | HCM |
| --- | --- | --- | --- | --- | --- | --- | --- | --- | --- |
| 1 | 1482.375 | 1120.175 | 2239.046 | 1370.17 | 2100.913 | 1695.572 | 2236.903 | 2025.183 | 1913.888 |
| 2 | 13376.567 | 13115.843 | 10744.596 | 12226.508 | 11795.2 | 12509.666 | 10998.973 | 11360.835 | 12209.266 |
| 3 | 18174.523 | 18153.782 | 18048.136 | 18085.391 | 18086.89 | 18148.403 | 18078.357 | 18057.993 | 18121.535 |
| 4 | 18450.729 | 18452.803 | 18433.172 | 18442.858 | 18439.301 | 18446.095 | 18436.669 | 18437.978 | 18447.063 |
| 5 | 18478.21 | 18479.245 | 18477.489 | 18478.125 | 18477.668 | 18478.37 | 18479.461 | 18477.134 | 18482.934 |
| 6 | 18483.312 | 18484.576 | 18482.341 | 18484.486 | 18483.342 | 18483.958 | 18484.093 | 18482.905 | 18488.248 |
| 7 | 18483.899 | 18485.094 | 18483.686 | 18485.039 | 18484.164 | 18484.655 | 18485.378 | 18483.798 | 18489.019 |
| 8 | 18484.085 | 18485.194 | 18483.809 | 18485.325 | 18484.265 | 18484.786 | 18485.432 | 18483.94 | 18489.235 |
| 9 | 18484.106 | 18485.196 | 18483.842 | 18485.333 | 18484.284 | 18484.79 | 18485.433 | 18483.965 | 18489.289 |
| 10 | 18484.113 | 18485.197 | 18483.842 | 18485.362 | 18484.284 | 18484.792 | 18485.433 | 18483.967 | 18489.289 |
| 11 | 18484.113 | 18485.197 | 18483.842 | 18485.368 | 18484.284 | 18484.792 | 18485.433 | 18483.967 | 18489.289 |
| 12 | 18484.113 | 18485.197 | 18483.842 | 18485.368 | 18484.284 | 18484.792 | 18485.433 | 18483.967 | 18489.289 |
| 13 | 18484.113 | 18485.197 | 18483.842 | 18485.368 | 18484.284 | 18484.792 | 18485.433 | 18483.967 | 18489.289 |
| 14 | 18484.113 | 18485.197 | 18483.842 | 18485.368 | 18484.284 | 18484.792 | 18485.433 | 18483.967 | 18489.289 |
| 15 | 18484.113 | 18485.197 | 18483.842 | 18485.368 | 18484.284 | 18484.792 | 18485.433 | 18483.967 | 18489.289 |
| 16 | 18484.113 | 18485.197 | 18483.842 | 18485.368 | 18484.284 | 18484.792 | 18485.433 | 18483.967 | 18489.289 |
| 17 | 18484.113 | 18485.197 | 18483.842 | 18485.368 | 18484.284 | 18484.792 | 18485.433 | 18483.967 | 18489.289 |
| 18 | 18484.113 | 18485.197 | 18483.842 | 18485.368 | 18484.284 | 18484.792 | 18485.433 | 18483.967 | 18489.289 |
| 19 | 18484.113 | 18485.197 | 18483.842 | 18485.368 | 18484.284 | 18484.792 | 18485.433 | 18483.967 | 18489.289 |
| 20 | 18484.113 | 18485.197 | 18483.842 | 18485.368 | 18484.284 | 18484.792 | 18485.433 | 18483.967 | 18489.289 |
| 21 | 18484.113 | 18485.197 | 18483.842 | 18485.368 | 18484.284 | 18484.792 | 18485.433 | 18483.967 | 18489.289 |
| 22 | 18484.113 | 18485.197 | 18483.842 | 18485.368 | 18484.284 | 18484.792 | 18485.433 | 18483.967 | 18489.289 |
| 23 | 18484.113 | 18485.197 | 18483.842 | 18485.368 | 18484.284 | 18484.792 | 18485.433 | 18483.967 | 18489.289 |
| 24 | 18484.113 | 18485.197 | 18483.842 | 18485.368 | 18484.284 | 18484.792 | 18485.433 | 18483.967 | 18489.289 |
| 25 | 18484.113 | 18485.197 | 18483.842 | 18485.368 | 18484.284 | 18484.792 | 18485.433 | 18483.967 | 18489.289 |
| 26 | 18484.113 | 18485.197 | 18483.842 | 18485.368 | 18484.284 | 18484.792 | 18485.433 | 18483.967 | 18489.289 |
| 27 | 18484.113 | 18485.197 | 18483.842 | 18485.368 | 18484.284 | 18484.792 | 18485.433 | 18483.967 | 18489.289 |
| 28 | 18484.113 | 18485.197 | 18483.842 | 18485.368 | 18484.284 | 18484.792 | 18485.433 | 18483.967 | 18489.289 |
| 29 | 18484.113 | 18485.197 | 18483.842 | 18485.368 | 18484.284 | 18484.792 | 18485.433 | 18483.967 | 18489.289 |
| 30 | 18484.113 | 18485.197 | 18483.842 | 18485.368 | 18484.284 | 18484.792 | 18485.433 | 18483.967 | 18489.289 |

**Tab S22. Infection values of the top ten nodes of the nine algorithms in EmailEU,** α **was set to 0.5,** *β* **was set to 1.**

| (a) David | BC | CC | DC | EC | GSI | GSM | ALSI | KBKNR | HCM |
| --- | --- | --- | --- | --- | --- | --- | --- | --- | --- |
| 1 | 62.931 | 60.719 | 62.181 | 59.125 | 61.638 | 61.659 | 61.619 | 61.783 | 61.877 |
| 2 | 89.281 | 87.59 | 87.8 | 86.201 | 87.194 | 87.356 | 87.302 | 87.349 | 87.529 |
| 3 | 94.494 | 94.021 | 94.166 | 93.76 | 93.971 | 93.947 | 94.149 | 93.974 | 94.178 |
| 4 | 95.235 | 95.088 | 95.16 | 94.947 | 95.12 | 95.035 | 95.249 | 95.098 | 95.336 |
| 5 | 95.32 | 95.218 | 95.249 | 95.08 | 95.239 | 95.156 | 95.363 | 95.233 | 95.483 |
| 6 | 95.327 | 95.235 | 95.26 | 95.098 | 95.262 | 95.175 | 95.375 | 95.245 | 95.5 |
| 7 | 95.328 | 95.238 | 95.261 | 95.099 | 95.265 | 95.176 | 95.376 | 95.245 | 95.504 |
| 8 | 95.328 | 95.238 | 95.261 | 95.099 | 95.266 | 95.176 | 95.376 | 95.245 | 95.504 |
| 9 | 95.328 | 95.238 | 95.261 | 95.099 | 95.266 | 95.176 | 95.376 | 95.245 | 95.504 |
| 10 | 95.328 | 95.238 | 95.261 | 95.099 | 95.266 | 95.176 | 95.376 | 95.245 | 95.504 |
| 11 | 95.328 | 95.238 | 95.261 | 95.099 | 95.266 | 95.176 | 95.376 | 95.245 | 95.504 |
| 12 | 95.328 | 95.238 | 95.261 | 95.099 | 95.266 | 95.176 | 95.376 | 95.245 | 95.504 |
| 13 | 95.328 | 95.238 | 95.261 | 95.099 | 95.266 | 95.176 | 95.376 | 95.245 | 95.504 |
| 14 | 95.328 | 95.238 | 95.261 | 95.099 | 95.266 | 95.176 | 95.376 | 95.245 | 95.504 |
| 15 | 95.328 | 95.238 | 95.261 | 95.099 | 95.266 | 95.176 | 95.376 | 95.245 | 95.504 |
| 16 | 95.328 | 95.238 | 95.261 | 95.099 | 95.266 | 95.176 | 95.376 | 95.245 | 95.504 |
| 17 | 95.328 | 95.238 | 95.261 | 95.099 | 95.266 | 95.176 | 95.376 | 95.245 | 95.504 |
| 18 | 95.328 | 95.238 | 95.261 | 95.099 | 95.266 | 95.176 | 95.376 | 95.245 | 95.504 |
| 19 | 95.328 | 95.238 | 95.261 | 95.099 | 95.266 | 95.176 | 95.376 | 95.245 | 95.504 |
| 20 | 95.328 | 95.238 | 95.261 | 95.099 | 95.266 | 95.176 | 95.376 | 95.245 | 95.504 |
| 21 | 95.328 | 95.238 | 95.261 | 95.099 | 95.266 | 95.176 | 95.376 | 95.245 | 95.504 |
| 22 | 95.328 | 95.238 | 95.261 | 95.099 | 95.266 | 95.176 | 95.376 | 95.245 | 95.504 |
| 23 | 95.328 | 95.238 | 95.261 | 95.099 | 95.266 | 95.176 | 95.376 | 95.245 | 95.504 |
| 24 | 95.328 | 95.238 | 95.261 | 95.099 | 95.266 | 95.176 | 95.376 | 95.245 | 95.504 |
| 25 | 95.328 | 95.238 | 95.261 | 95.099 | 95.266 | 95.176 | 95.376 | 95.245 | 95.504 |
| 26 | 95.328 | 95.238 | 95.261 | 95.099 | 95.266 | 95.176 | 95.376 | 95.245 | 95.504 |
| 27 | 95.328 | 95.238 | 95.261 | 95.099 | 95.266 | 95.176 | 95.376 | 95.245 | 95.504 |
| 28 | 95.328 | 95.238 | 95.261 | 95.099 | 95.266 | 95.176 | 95.376 | 95.245 | 95.504 |
| 29 | 95.328 | 95.238 | 95.261 | 95.099 | 95.266 | 95.176 | 95.376 | 95.245 | 95.504 |
| 30 | 95.328 | 95.238 | 95.261 | 95.099 | 95.266 | 95.176 | 95.376 | 95.245 | 95.504 |

**Tab S23. Infection values of the top ten nodes of the nine algorithms in David,** α **was set to 0.4,** *β* **was set to 1.**

| (b) Netscience | BC | CC | DC | EC | GSI | GSM | ALSI | KBKNR | HCM |
| --- | --- | --- | --- | --- | --- | --- | --- | --- | --- |
| 1 | 60.6 | 51.968 | 76.214 | 31.739 | 68.841 | 66.387 | 76.35 | 39.526 | 76.899 |
| 2 | 115.715 | 102.555 | 133.333 | 48.239 | 115.177 | 114.327 | 133.669 | 66.554 | 134.861 |
| 3 | 159.699 | 142.76 | 173.032 | 64.339 | 152.219 | 149.492 | 173.539 | 91.178 | 175.424 |
| 4 | 188.408 | 169.243 | 198.222 | 79.561 | 178.829 | 171.204 | 198.293 | 113.542 | 200.68 |
| 5 | 204.517 | 185.406 | 211.916 | 92.909 | 195.658 | 183.915 | 211.689 | 133.091 | 214.217 |
| 6 | 212.884 | 195.07 | 218.339 | 104.672 | 206.039 | 191.554 | 218.044 | 150.239 | 220.722 |
| 7 | 217.054 | 201.192 | 221.228 | 115.045 | 212.366 | 196.435 | 221.012 | 164.809 | 223.583 |
| 8 | 219.109 | 204.993 | 222.43 | 124.045 | 215.974 | 199.492 | 222.27 | 175.782 | 224.776 |
| 9 | 220.064 | 207.149 | 222.946 | 131.558 | 217.958 | 201.343 | 222.819 | 183.281 | 225.27 |
| 10 | 220.543 | 208.359 | 223.132 | 137.526 | 219.012 | 202.381 | 223.04 | 188.17 | 225.457 |
| 11 | 220.796 | 209.036 | 223.212 | 142.088 | 219.552 | 202.932 | 223.136 | 191.045 | 225.522 |
| 12 | 220.924 | 209.333 | 223.251 | 145.6 | 219.799 | 203.225 | 223.188 | 192.854 | 225.547 |
| 13 | 220.976 | 209.48 | 223.276 | 148.16 | 219.911 | 203.354 | 223.207 | 194.008 | 225.559 |
| 14 | 221.005 | 209.544 | 223.287 | 150.033 | 219.951 | 203.409 | 223.218 | 194.672 | 225.563 |
| 15 | 221.022 | 209.564 | 223.29 | 151.33 | 219.973 | 203.436 | 223.222 | 195.092 | 225.564 |
| 16 | 221.032 | 209.569 | 223.292 | 152.185 | 219.98 | 203.444 | 223.222 | 195.333 | 225.564 |
| 17 | 221.037 | 209.57 | 223.292 | 152.725 | 219.985 | 203.446 | 223.222 | 195.467 | 225.564 |
| 18 | 221.038 | 209.57 | 223.292 | 153.073 | 219.986 | 203.446 | 223.222 | 195.561 | 225.564 |
| 19 | 221.039 | 209.57 | 223.292 | 153.275 | 219.987 | 203.446 | 223.222 | 195.631 | 225.564 |
| 20 | 221.039 | 209.57 | 223.292 | 153.392 | 219.988 | 203.446 | 223.222 | 195.675 | 225.564 |
| 21 | 221.039 | 209.57 | 223.292 | 153.461 | 219.988 | 203.446 | 223.222 | 195.713 | 225.564 |
| 22 | 221.039 | 209.57 | 223.292 | 153.497 | 219.988 | 203.446 | 223.222 | 195.729 | 225.564 |
| 23 | 221.039 | 209.57 | 223.292 | 153.51 | 219.988 | 203.446 | 223.222 | 195.74 | 225.564 |
| 24 | 221.039 | 209.57 | 223.292 | 153.52 | 219.988 | 203.446 | 223.222 | 195.741 | 225.564 |
| 25 | 221.039 | 209.57 | 223.292 | 153.522 | 219.988 | 203.446 | 223.222 | 195.741 | 225.564 |
| 26 | 221.039 | 209.57 | 223.292 | 153.522 | 219.988 | 203.446 | 223.222 | 195.741 | 225.564 |
| 27 | 221.039 | 209.57 | 223.292 | 153.522 | 219.988 | 203.446 | 223.222 | 195.741 | 225.564 |
| 28 | 221.039 | 209.57 | 223.292 | 153.522 | 219.988 | 203.446 | 223.222 | 195.741 | 225.564 |
| 29 | 221.039 | 209.57 | 223.292 | 153.522 | 219.988 | 203.446 | 223.222 | 195.741 | 225.564 |
| 30 | 221.039 | 209.57 | 223.292 | 153.522 | 219.988 | 203.446 | 223.222 | 195.741 | 225.564 |

**Tab S24. Infection values of the top ten nodes of the nine algorithms in Netscience,** α **was set to 0.4,** *β* **was set to 1.**

| (c) Hamsterster | BC | CC | DC | EC | GSI | GSM | ALSI | KBKNR | HCM |
| --- | --- | --- | --- | --- | --- | --- | --- | --- | --- |
| 1 | 452.366 | 375.513 | 449.866 | 360.119 | 409.591 | 415.41 | 449.69 | 58.051 | 409.977 |
| 2 | 1309.79 | 1179.673 | 1222.454 | 1042.563 | 1129.571 | 1185.176 | 1222.713 | 290.125 | 1131.321 |
| 3 | 1634.352 | 1590.325 | 1608.802 | 1522.457 | 1567.675 | 1589.41 | 1608.478 | 1133.878 | 1569.246 |
| 4 | 1708.983 | 1695.474 | 1700.885 | 1682.89 | 1691.301 | 1695.743 | 1699.813 | 1585.025 | 1692.746 |
| 5 | 1729.08 | 1725.502 | 1726.325 | 1721.916 | 1723.332 | 1725.485 | 1725.526 | 1698.382 | 1724.369 |
| 6 | 1734.264 | 1733.461 | 1733.72 | 1732.456 | 1732.176 | 1733.256 | 1732.815 | 1727.158 | 1733.119 |
| 7 | 1735.474 | 1735.362 | 1735.727 | 1735.046 | 1734.561 | 1735.137 | 1734.768 | 1733.339 | 1735.431 |
| 8 | 1735.708 | 1735.716 | 1736.209 | 1735.639 | 1735.126 | 1735.482 | 1735.262 | 1734.584 | 1735.998 |
| 9 | 1735.744 | 1735.787 | 1736.316 | 1735.743 | 1735.244 | 1735.544 | 1735.373 | 1734.775 | 1736.106 |
| 10 | 1735.755 | 1735.796 | 1736.337 | 1735.759 | 1735.258 | 1735.549 | 1735.396 | 1734.807 | 1736.128 |
| 11 | 1735.759 | 1735.798 | 1736.34 | 1735.761 | 1735.262 | 1735.549 | 1735.399 | 1734.808 | 1736.133 |
| 12 | 1735.761 | 1735.798 | 1736.341 | 1735.761 | 1735.262 | 1735.549 | 1735.401 | 1734.808 | 1736.133 |
| 13 | 1735.761 | 1735.798 | 1736.341 | 1735.761 | 1735.262 | 1735.549 | 1735.401 | 1734.808 | 1736.133 |
| 14 | 1735.761 | 1735.798 | 1736.341 | 1735.761 | 1735.262 | 1735.549 | 1735.401 | 1734.808 | 1736.133 |
| 15 | 1735.761 | 1735.798 | 1736.341 | 1735.761 | 1735.262 | 1735.549 | 1735.401 | 1734.808 | 1736.133 |
| 16 | 1735.761 | 1735.798 | 1736.341 | 1735.761 | 1735.262 | 1735.549 | 1735.401 | 1734.808 | 1736.133 |
| 17 | 1735.761 | 1735.798 | 1736.341 | 1735.761 | 1735.262 | 1735.549 | 1735.401 | 1734.808 | 1736.133 |
| 18 | 1735.761 | 1735.798 | 1736.341 | 1735.761 | 1735.262 | 1735.549 | 1735.401 | 1734.808 | 1736.133 |
| 19 | 1735.761 | 1735.798 | 1736.341 | 1735.761 | 1735.262 | 1735.549 | 1735.401 | 1734.808 | 1736.133 |
| 20 | 1735.761 | 1735.798 | 1736.341 | 1735.761 | 1735.262 | 1735.549 | 1735.401 | 1734.808 | 1736.133 |
| 21 | 1735.761 | 1735.798 | 1736.341 | 1735.761 | 1735.262 | 1735.549 | 1735.401 | 1734.808 | 1736.133 |
| 22 | 1735.761 | 1735.798 | 1736.341 | 1735.761 | 1735.262 | 1735.549 | 1735.401 | 1734.808 | 1736.133 |
| 23 | 1735.761 | 1735.798 | 1736.341 | 1735.761 | 1735.262 | 1735.549 | 1735.401 | 1734.808 | 1736.133 |
| 24 | 1735.761 | 1735.798 | 1736.341 | 1735.761 | 1735.262 | 1735.549 | 1735.401 | 1734.808 | 1736.133 |
| 25 | 1735.761 | 1735.798 | 1736.341 | 1735.761 | 1735.262 | 1735.549 | 1735.401 | 1734.808 | 1736.133 |
| 26 | 1735.761 | 1735.798 | 1736.341 | 1735.761 | 1735.262 | 1735.549 | 1735.401 | 1734.808 | 1736.133 |
| 27 | 1735.761 | 1735.798 | 1736.341 | 1735.761 | 1735.262 | 1735.549 | 1735.401 | 1734.808 | 1736.133 |
| 28 | 1735.761 | 1735.798 | 1736.341 | 1735.761 | 1735.262 | 1735.549 | 1735.401 | 1734.808 | 1736.133 |
| 29 | 1735.761 | 1735.798 | 1736.341 | 1735.761 | 1735.262 | 1735.549 | 1735.401 | 1734.808 | 1736.133 |
| 30 | 1735.761 | 1735.798 | 1736.341 | 1735.761 | 1735.262 | 1735.549 | 1735.401 | 1734.808 | 1736.133 |

**Tab S25. Infection values of the top ten nodes of the nine algorithms in Hamsterster,** α **was set to 0.4,** *β* **was set to 1.**

| (d) Ca-GrQc | BC | CC | DC | EC | GSI | GSM | ALSI | KBKNR | HCM |
| --- | --- | --- | --- | --- | --- | --- | --- | --- | --- |
| 1 | 150.384 | 152.951 | 130.043 | 100.64 | 130.059 | 127.505 | 130.374 | 130.482 | 130.212 |
| 2 | 522.839 | 473.954 | 315.543 | 259.622 | 314.781 | 315.922 | 315.925 | 316.437 | 315.992 |
| 3 | 1093.206 | 967.438 | 605.154 | 521.158 | 605.971 | 609.955 | 605.195 | 607.649 | 607.678 |
| 4 | 1685.146 | 1541.979 | 1031.256 | 909.566 | 1035.539 | 1038.202 | 1033.232 | 1035.681 | 1037.41 |
| 5 | 2122.861 | 2019.463 | 1551.333 | 1413.438 | 1557.744 | 1558.064 | 1553.605 | 1557.784 | 1560.972 |
| 6 | 2388.119 | 2329.232 | 2012.928 | 1902.698 | 2019.116 | 2015.932 | 2015.224 | 2018.602 | 2022.983 |
| 7 | 2526.957 | 2495.905 | 2320.511 | 2253.814 | 2324.041 | 2320.213 | 2322.257 | 2323.956 | 2327.156 |
| 8 | 2591.144 | 2575.506 | 2489.473 | 2454.042 | 2491.619 | 2487.658 | 2489.892 | 2491.686 | 2492.316 |
| 9 | 2619.695 | 2611.381 | 2571.351 | 2553.15 | 2573.584 | 2569.734 | 2570.549 | 2573.642 | 2573.152 |
| 10 | 2632.188 | 2627.68 | 2608.947 | 2599.319 | 2611.348 | 2607.462 | 2607.932 | 2611.625 | 2611.027 |
| 11 | 2637.841 | 2635.064 | 2625.941 | 2620.771 | 2628.482 | 2624.421 | 2625.096 | 2628.912 | 2628.496 |
| 12 | 2640.279 | 2638.331 | 2633.63 | 2630.739 | 2636.233 | 2632.04 | 2633.024 | 2636.645 | 2636.449 |
| 13 | 2641.368 | 2639.777 | 2637.054 | 2635.263 | 2639.863 | 2635.489 | 2636.701 | 2640.159 | 2640.108 |
| 14 | 2641.857 | 2640.44 | 2638.585 | 2637.376 | 2641.5 | 2637.056 | 2638.359 | 2641.806 | 2641.782 |
| 15 | 2642.057 | 2640.728 | 2639.319 | 2638.306 | 2642.252 | 2637.786 | 2639.076 | 2642.547 | 2642.515 |
| 16 | 2642.139 | 2640.852 | 2639.651 | 2638.74 | 2642.607 | 2638.122 | 2639.381 | 2642.868 | 2642.863 |
| 17 | 2642.178 | 2640.906 | 2639.784 | 2638.925 | 2642.773 | 2638.259 | 2639.507 | 2643.018 | 2643.001 |
| 18 | 2642.192 | 2640.926 | 2639.833 | 2639.011 | 2642.853 | 2638.311 | 2639.558 | 2643.105 | 2643.067 |
| 19 | 2642.196 | 2640.933 | 2639.855 | 2639.037 | 2642.877 | 2638.34 | 2639.573 | 2643.129 | 2643.085 |
| 20 | 2642.199 | 2640.938 | 2639.868 | 2639.049 | 2642.9 | 2638.344 | 2639.578 | 2643.137 | 2643.094 |
| 21 | 2642.2 | 2640.94 | 2639.871 | 2639.06 | 2642.913 | 2638.345 | 2639.581 | 2643.143 | 2643.1 |
| 22 | 2642.2 | 2640.946 | 2639.873 | 2639.064 | 2642.917 | 2638.346 | 2639.587 | 2643.146 | 2643.432 |
| 23 | 2642.2 | 2640.949 | 2639.874 | 2639.064 | 2642.918 | 2638.346 | 2639.59 | 2643.146 | 2643.432 |
| 24 | 2642.2 | 2640.949 | 2639.875 | 2639.064 | 2642.918 | 2638.346 | 2639.591 | 2643.146 | 2643.432 |
| 25 | 2642.2 | 2640.949 | 2639.875 | 2639.064 | 2642.918 | 2638.346 | 2639.591 | 2643.146 | 2643.432 |
| 26 | 2642.2 | 2640.949 | 2639.875 | 2639.064 | 2642.918 | 2638.346 | 2639.591 | 2643.146 | 2643.432 |
| 27 | 2642.2 | 2640.949 | 2639.875 | 2639.064 | 2642.918 | 2638.346 | 2639.591 | 2643.146 | 2643.432 |
| 28 | 2642.2 | 2640.949 | 2639.875 | 2639.064 | 2642.918 | 2638.346 | 2639.591 | 2643.146 | 2643.432 |
| 29 | 2642.2 | 2640.949 | 2639.875 | 2639.064 | 2642.918 | 2638.346 | 2639.591 | 2643.146 | 2643.432 |
| 30 | 2642.2 | 2640.949 | 2639.875 | 2639.064 | 2642.918 | 2638.346 | 2639.591 | 2643.146 | 2643.432 |

**Tab S26. Infection values of the top ten nodes of the nine algorithms in Ca-GrQc,** α **was set to 0.4,** *β* **was set to 1.**

| (e) AS | BC | CC | DC | EC | GSI | GSM | ALSI | KBKNR | HCM |
| --- | --- | --- | --- | --- | --- | --- | --- | --- | --- |
| 1 | 1477.617 | 1438.977 | 1540.012 | 1478.715 | 1515.876 | 1425.895 | 1540.397 | 1515.938 | 1516.1 |
| 2 | 2824.25 | 2757.51 | 2672.499 | 2653.82 | 2699.175 | 2759.593 | 2675.224 | 2702.465 | 2703.164 |
| 3 | 3399.982 | 3361.799 | 3322.377 | 3304.474 | 3335.409 | 3360.716 | 3323.115 | 3335.559 | 3334.663 |
| 4 | 3584.007 | 3574.221 | 3561.265 | 3554.518 | 3566.016 | 3573.865 | 3561.347 | 3565.8 | 3565.261 |
| 5 | 3633.029 | 3631.16 | 3627.086 | 3624.258 | 3628.357 | 3629.895 | 3627.046 | 3627.153 | 3628.055 |
| 6 | 3646.444 | 3646.962 | 3645.34 | 3644.093 | 3645.721 | 3645.752 | 3645.194 | 3644.486 | 3646.291 |
| 7 | 3650.407 | 3651.039 | 3650.144 | 3649.663 | 3650.421 | 3650.011 | 3650.066 | 3648.949 | 3651.174 |
| 8 | 3651.451 | 3652.324 | 3651.545 | 3651.462 | 3651.88 | 3651.3 | 3651.695 | 3650.477 | 3652.591 |
| 9 | 3651.762 | 3652.683 | 3651.981 | 3652.001 | 3652.357 | 3651.642 | 3652.164 | 3650.847 | 3652.968 |
| 10 | 3651.863 | 3652.766 | 3652.095 | 3652.146 | 3652.49 | 3651.739 | 3652.285 | 3650.968 | 3653.093 |
| 11 | 3651.903 | 3652.784 | 3652.142 | 3652.198 | 3652.52 | 3651.766 | 3652.355 | 3650.999 | 3653.147 |
| 12 | 3651.919 | 3652.795 | 3652.148 | 3652.224 | 3652.53 | 3651.771 | 3652.362 | 3651.007 | 3653.152 |
| 13 | 3651.92 | 3652.796 | 3652.155 | 3652.228 | 3652.534 | 3651.771 | 3652.369 | 3651.007 | 3653.158 |
| 14 | 3651.92 | 3652.796 | 3652.156 | 3652.228 | 3652.535 | 3651.771 | 3652.369 | 3651.007 | 3653.159 |
| 15 | 3651.92 | 3652.796 | 3652.156 | 3652.228 | 3652.535 | 3651.771 | 3652.369 | 3651.007 | 3653.159 |
| 16 | 3651.92 | 3652.796 | 3652.156 | 3652.228 | 3652.535 | 3651.771 | 3652.369 | 3651.007 | 3653.159 |
| 17 | 3651.92 | 3652.796 | 3652.156 | 3652.228 | 3652.535 | 3651.771 | 3652.369 | 3651.007 | 3653.159 |
| 18 | 3651.92 | 3652.796 | 3652.156 | 3652.228 | 3652.535 | 3651.771 | 3652.369 | 3651.007 | 3653.159 |
| 19 | 3651.92 | 3652.796 | 3652.156 | 3652.228 | 3652.535 | 3651.771 | 3652.369 | 3651.007 | 3653.159 |
| 20 | 3651.92 | 3652.796 | 3652.156 | 3652.228 | 3652.535 | 3651.771 | 3652.369 | 3651.007 | 3653.159 |
| 21 | 3651.92 | 3652.796 | 3652.156 | 3652.228 | 3652.535 | 3651.771 | 3652.369 | 3651.007 | 3653.159 |
| 22 | 3651.92 | 3652.796 | 3652.156 | 3652.228 | 3652.535 | 3651.771 | 3652.369 | 3651.007 | 3653.159 |
| 23 | 3651.92 | 3652.796 | 3652.156 | 3652.228 | 3652.535 | 3651.771 | 3652.369 | 3651.007 | 3653.159 |
| 24 | 3651.92 | 3652.796 | 3652.156 | 3652.228 | 3652.535 | 3651.771 | 3652.369 | 3651.007 | 3653.159 |
| 25 | 3651.92 | 3652.796 | 3652.156 | 3652.228 | 3652.535 | 3651.771 | 3652.369 | 3651.007 | 3653.159 |
| 26 | 3651.92 | 3652.796 | 3652.156 | 3652.228 | 3652.535 | 3651.771 | 3652.369 | 3651.007 | 3653.159 |
| 27 | 3651.92 | 3652.796 | 3652.156 | 3652.228 | 3652.535 | 3651.771 | 3652.369 | 3651.007 | 3653.159 |
| 28 | 3651.92 | 3652.796 | 3652.156 | 3652.228 | 3652.535 | 3651.771 | 3652.369 | 3651.007 | 3653.159 |
| 29 | 3651.92 | 3652.796 | 3652.156 | 3652.228 | 3652.535 | 3651.771 | 3652.369 | 3651.007 | 3653.159 |
| 30 | 3651.92 | 3652.796 | 3652.156 | 3652.228 | 3652.535 | 3651.771 | 3652.369 | 3651.007 | 3653.159 |

**Tab S27. Infection values of the top ten nodes of the nine algorithms in AS,** α **was set to 0.4,** *β* **was set to 1.**

| (f) Lastfm | BC | CC | DC | EC | GSI | GSM | ALSI | KBKNR | HCM |
| --- | --- | --- | --- | --- | --- | --- | --- | --- | --- |
| 1 | 451.743 | 412.66 | 573.388 | 220.36 | 391.799 | 204.304 | 572.878 | 272.393 | 491.354 |
| 2 | 1816.134 | 1666.315 | 2006.93 | 712.149 | 1295.37 | 672.806 | 2004.588 | 785.021 | 1630.711 |
| 3 | 3693.251 | 3512.926 | 3664.268 | 1642.228 | 2752.235 | 1611.824 | 3660.165 | 1801.286 | 3163.689 |
| 4 | 4752.113 | 4673.93 | 4650.986 | 3135.795 | 4125.744 | 3074.46 | 4648.667 | 3324.919 | 4368.879 |
| 5 | 5165.146 | 5143.019 | 5101.412 | 4390.374 | 4885.872 | 4342.664 | 5100.266 | 4498.067 | 4991.287 |
| 6 | 5304.939 | 5298.849 | 5277.301 | 5006.925 | 5200.23 | 4985.005 | 5275.594 | 5048.403 | 5240.499 |
| 7 | 5349.849 | 5347.672 | 5340.695 | 5246.855 | 5314.472 | 5239.044 | 5338.023 | 5260.651 | 5329.628 |
| 8 | 5364.192 | 5363.11 | 5362.433 | 5330.348 | 5353.656 | 5327.998 | 5359.433 | 5334.44 | 5359.803 |
| 9 | 5368.728 | 5367.928 | 5369.717 | 5358.172 | 5366.445 | 5357.454 | 5366.614 | 5359.193 | 5369.821 |
| 10 | 5370.138 | 5369.441 | 5372.082 | 5366.889 | 5370.528 | 5367.075 | 5368.791 | 5367.401 | 5373.068 |
| 11 | 5370.589 | 5369.921 | 5372.854 | 5369.677 | 5371.821 | 5370.088 | 5369.474 | 5369.897 | 5374.089 |
| 12 | 5370.732 | 5370.07 | 5373.064 | 5370.538 | 5372.21 | 5371.055 | 5369.685 | 5370.675 | 5374.449 |
| 13 | 5370.784 | 5370.133 | 5373.118 | 5370.8 | 5372.345 | 5371.322 | 5369.752 | 5370.931 | 5374.552 |
| 14 | 5370.802 | 5370.155 | 5373.134 | 5370.889 | 5372.383 | 5371.405 | 5369.773 | 5371.025 | 5374.584 |
| 15 | 5370.806 | 5370.158 | 5373.14 | 5370.925 | 5372.398 | 5371.434 | 5369.778 | 5371.056 | 5374.598 |
| 16 | 5370.807 | 5370.158 | 5373.142 | 5370.932 | 5372.402 | 5371.438 | 5369.779 | 5371.061 | 5374.599 |
| 17 | 5370.807 | 5370.158 | 5373.142 | 5370.936 | 5372.404 | 5371.439 | 5369.779 | 5371.064 | 5374.6 |
| 18 | 5370.807 | 5370.158 | 5373.142 | 5370.936 | 5372.405 | 5371.441 | 5369.779 | 5371.069 | 5374.6 |
| 19 | 5370.807 | 5370.158 | 5373.142 | 5370.936 | 5372.405 | 5371.441 | 5369.779 | 5371.071 | 5374.6 |
| 20 | 5370.807 | 5370.158 | 5373.142 | 5370.936 | 5372.405 | 5371.441 | 5369.779 | 5371.072 | 5374.6 |
| 21 | 5370.807 | 5370.158 | 5373.142 | 5370.936 | 5372.405 | 5371.441 | 5369.779 | 5371.076 | 5374.6 |
| 22 | 5370.807 | 5370.158 | 5373.142 | 5370.936 | 5372.405 | 5371.441 | 5369.779 | 5371.076 | 5374.6 |
| 23 | 5370.807 | 5370.158 | 5373.142 | 5370.936 | 5372.405 | 5371.441 | 5369.779 | 5371.076 | 5374.6 |
| 24 | 5370.807 | 5370.158 | 5373.142 | 5370.936 | 5372.405 | 5371.441 | 5369.779 | 5371.076 | 5374.6 |
| 25 | 5370.807 | 5370.158 | 5373.142 | 5370.936 | 5372.405 | 5371.441 | 5369.779 | 5371.076 | 5374.6 |
| 26 | 5370.807 | 5370.158 | 5373.142 | 5370.936 | 5372.405 | 5371.441 | 5369.779 | 5371.076 | 5374.6 |
| 27 | 5370.807 | 5370.158 | 5373.142 | 5370.936 | 5372.405 | 5371.441 | 5369.779 | 5371.076 | 5374.6 |
| 28 | 5370.807 | 5370.158 | 5373.142 | 5370.936 | 5372.405 | 5371.441 | 5369.779 | 5371.076 | 5374.6 |
| 29 | 5370.807 | 5370.158 | 5373.142 | 5370.936 | 5372.405 | 5371.441 | 5369.779 | 5371.076 | 5374.6 |
| 30 | 5370.807 | 5370.158 | 5373.142 | 5370.936 | 5372.405 | 5371.441 | 5369.779 | 5371.076 | 5374.6 |

**Tab S28. Infection values of the top ten nodes of the nine algorithms in Lastfm,** α **was set to 0.4,** *β* **was set to 1.**

| (g) Dblp | BC | CC | DC | EC | GSI | GSM | ALSI | KBKNR | HCM |
| --- | --- | --- | --- | --- | --- | --- | --- | --- | --- |
| 1 | 1269.344 | 1265.081 | 1267.6 | 1197.143 | 1283.362 | 1262.197 | 1282.317 | 1281.042 | 1282.624 |
| 2 | 4599.034 | 4474.519 | 4461.846 | 4304.712 | 4531.416 | 4442.377 | 4524.861 | 4522.996 | 4522.427 |
| 3 | 7543.422 | 7551.868 | 7532.798 | 7375.013 | 7546.068 | 7546.108 | 7545.602 | 7543.973 | 7544.33 |
| 4 | 8307.764 | 8301.807 | 8301.156 | 8269.938 | 8304.652 | 8299.309 | 8302.116 | 8303.182 | 8305.393 |
| 5 | 8465.188 | 8467.081 | 8467.488 | 8456.454 | 8467.358 | 8466.458 | 8464.965 | 8466.842 | 8468.676 |
| 6 | 8490.906 | 8492.721 | 8492.332 | 8490.39 | 8492.552 | 8491.989 | 8490.555 | 8493.035 | 8494.198 |
| 7 | 8496.104 | 8498.1 | 8497.886 | 8496.776 | 8497.844 | 8497.372 | 8495.963 | 8498.536 | 8499.522 |
| 8 | 8496.976 | 8499.058 | 8498.774 | 8498.081 | 8498.764 | 8498.387 | 8496.93 | 8499.617 | 8500.357 |
| 9 | 8497.216 | 8499.287 | 8498.94 | 8498.319 | 8499.038 | 8498.606 | 8497.212 | 8499.797 | 8500.58 |
| 10 | 8497.266 | 8499.341 | 8498.978 | 8498.39 | 8499.072 | 8498.635 | 8497.283 | 8499.84 | 8500.637 |
| 11 | 8497.28 | 8499.347 | 8498.98 | 8498.395 | 8499.076 | 8498.638 | 8497.297 | 8499.872 | 8500.654 |
| 12 | 8497.28 | 8499.349 | 8498.98 | 8498.395 | 8499.076 | 8498.643 | 8497.298 | 8499.874 | 8500.665 |
| 13 | 8497.28 | 8499.349 | 8498.98 | 8498.395 | 8499.076 | 8498.643 | 8497.298 | 8499.874 | 8500.666 |
| 14 | 8497.28 | 8499.349 | 8498.98 | 8498.395 | 8499.076 | 8498.643 | 8497.298 | 8499.874 | 8500.667 |
| 15 | 8497.28 | 8499.349 | 8498.98 | 8498.395 | 8499.076 | 8498.643 | 8497.298 | 8499.874 | 8500.667 |
| 16 | 8497.28 | 8499.349 | 8498.98 | 8498.395 | 8499.076 | 8498.643 | 8497.298 | 8499.874 | 8500.667 |
| 17 | 8497.28 | 8499.349 | 8498.98 | 8498.395 | 8499.076 | 8498.643 | 8497.298 | 8499.874 | 8500.667 |
| 18 | 8497.28 | 8499.349 | 8498.98 | 8498.395 | 8499.076 | 8498.643 | 8497.298 | 8499.874 | 8500.667 |
| 19 | 8497.28 | 8499.349 | 8498.98 | 8498.395 | 8499.076 | 8498.643 | 8497.298 | 8499.874 | 8500.667 |
| 20 | 8497.28 | 8499.349 | 8498.98 | 8498.395 | 8499.076 | 8498.643 | 8497.298 | 8499.874 | 8500.667 |
| 21 | 8497.28 | 8499.349 | 8498.98 | 8498.395 | 8499.076 | 8498.643 | 8497.298 | 8499.874 | 8500.667 |
| 22 | 8497.28 | 8499.349 | 8498.98 | 8498.395 | 8499.076 | 8498.643 | 8497.298 | 8499.874 | 8500.667 |
| 23 | 8497.28 | 8499.349 | 8498.98 | 8498.395 | 8499.076 | 8498.643 | 8497.298 | 8499.874 | 8500.667 |
| 24 | 8497.28 | 8499.349 | 8498.98 | 8498.395 | 8499.076 | 8498.643 | 8497.298 | 8499.874 | 8500.667 |
| 25 | 8497.28 | 8499.349 | 8498.98 | 8498.395 | 8499.076 | 8498.643 | 8497.298 | 8499.874 | 8500.667 |
| 26 | 8497.28 | 8499.349 | 8498.98 | 8498.395 | 8499.076 | 8498.643 | 8497.298 | 8499.874 | 8500.667 |
| 27 | 8497.28 | 8499.349 | 8498.98 | 8498.395 | 8499.076 | 8498.643 | 8497.298 | 8499.874 | 8500.667 |
| 28 | 8497.28 | 8499.349 | 8498.98 | 8498.395 | 8499.076 | 8498.643 | 8497.298 | 8499.874 | 8500.667 |
| 29 | 8497.28 | 8499.349 | 8498.98 | 8498.395 | 8499.076 | 8498.643 | 8497.298 | 8499.874 | 8500.667 |
| 30 | 8497.28 | 8499.349 | 8498.98 | 8498.395 | 8499.076 | 8498.643 | 8497.298 | 8499.874 | 8500.667 |

**Tab S29. Infection values of the top ten nodes of the nine algorithms in Dblp,** α **was set to 0.4,** *β* **was set to 1.**

| (h) Ca-Astroph | BC | CC | DC | EC | GSI | GSM | ALSI | KBKNR | HCM |
| --- | --- | --- | --- | --- | --- | --- | --- | --- | --- |
| 1 | 1061.057 | 1060.507 | 1103.69 | 787.927 | 1023.55 | 1100.714 | 1103.876 | 587.824 | 987.109 |
| 2 | 6934.008 | 6519.116 | 6608.466 | 5386.449 | 6253.597 | 6634.842 | 6611.299 | 4812.7 | 6146.836 |
| 3 | 12777.565 | 12381.654 | 12434.743 | 11759.093 | 12240.302 | 12445.454 | 12437.229 | 11434.938 | 12178.208 |
| 4 | 14866.863 | 14714.867 | 14726.957 | 14508.523 | 14661.448 | 14734.868 | 14729.06 | 14426.516 | 14642.872 |
| 5 | 15456.744 | 15412.587 | 15412.909 | 15352.497 | 15394.952 | 15417.084 | 15413.521 | 15332.588 | 15392.431 |
| 6 | 15618.152 | 15605.596 | 15603.223 | 15588.081 | 15598.79 | 15605.312 | 15602.935 | 15584.62 | 15600.801 |
| 7 | 15667.882 | 15663.893 | 15661.579 | 15657.055 | 15659.984 | 15662.802 | 15660.965 | 15657.998 | 15662.381 |
| 8 | 15684.775 | 15684.002 | 15681.513 | 15680.159 | 15680.981 | 15682.56 | 15680.849 | 15682.204 | 15683.442 |
| 9 | 15690.694 | 15691.143 | 15688.555 | 15688.062 | 15688.444 | 15689.47 | 15688.01 | 15690.552 | 15690.899 |
| 10 | 15692.524 | 15693.612 | 15690.917 | 15690.774 | 15690.922 | 15691.796 | 15690.359 | 15693.422 | 15693.495 |
| 11 | 15693.101 | 15694.426 | 15691.652 | 15691.59 | 15691.733 | 15692.512 | 15691.091 | 15694.284 | 15694.32 |
| 12 | 15693.232 | 15694.635 | 15691.858 | 15691.824 | 15691.934 | 15692.768 | 15691.31 | 15694.534 | 15694.551 |
| 13 | 15693.266 | 15694.688 | 15691.915 | 15691.872 | 15691.977 | 15692.826 | 15691.354 | 15694.598 | 15694.615 |
| 14 | 15693.274 | 15694.699 | 15691.934 | 15691.881 | 15691.994 | 15692.844 | 15691.364 | 15694.614 | 15694.627 |
| 15 | 15693.275 | 15694.702 | 15691.939 | 15691.882 | 15691.996 | 15692.848 | 15691.365 | 15694.62 | 15694.931 |
| 16 | 15693.276 | 15694.702 | 15691.94 | 15691.882 | 15691.996 | 15692.848 | 15691.365 | 15694.62 | 15694.931 |
| 17 | 15693.276 | 15694.702 | 15691.94 | 15691.882 | 15691.996 | 15692.848 | 15691.365 | 15694.62 | 15694.931 |
| 18 | 15693.276 | 15694.702 | 15691.94 | 15691.882 | 15691.996 | 15692.848 | 15691.365 | 15694.62 | 15694.931 |
| 19 | 15693.276 | 15694.702 | 15691.94 | 15691.882 | 15691.996 | 15692.848 | 15691.365 | 15694.62 | 15694.931 |
| 20 | 15693.276 | 15694.702 | 15691.94 | 15691.882 | 15691.996 | 15692.848 | 15691.365 | 15694.62 | 15694.931 |
| 21 | 15693.276 | 15694.702 | 15691.94 | 15691.882 | 15691.996 | 15692.848 | 15691.365 | 15694.62 | 15694.931 |
| 22 | 15693.276 | 15694.702 | 15691.94 | 15691.882 | 15691.996 | 15692.848 | 15691.365 | 15694.62 | 15694.931 |
| 23 | 15693.276 | 15694.702 | 15691.94 | 15691.882 | 15691.996 | 15692.848 | 15691.365 | 15694.62 | 15694.931 |
| 24 | 15693.276 | 15694.702 | 15691.94 | 15691.882 | 15691.996 | 15692.848 | 15691.365 | 15694.62 | 15694.931 |
| 25 | 15693.276 | 15694.702 | 15691.94 | 15691.882 | 15691.996 | 15692.848 | 15691.365 | 15694.62 | 15694.931 |
| 26 | 15693.276 | 15694.702 | 15691.94 | 15691.882 | 15691.996 | 15692.848 | 15691.365 | 15694.62 | 15694.931 |
| 27 | 15693.276 | 15694.702 | 15691.94 | 15691.882 | 15691.996 | 15692.848 | 15691.365 | 15694.62 | 15694.931 |
| 28 | 15693.276 | 15694.702 | 15691.94 | 15691.882 | 15691.996 | 15692.848 | 15691.365 | 15694.62 | 15694.931 |
| 29 | 15693.276 | 15694.702 | 15691.94 | 15691.882 | 15691.996 | 15692.848 | 15691.365 | 15694.62 | 15694.931 |
| 30 | 15693.276 | 15694.702 | 15691.94 | 15691.882 | 15691.996 | 15692.848 | 15691.365 | 15694.62 | 15694.931 |

**Tab S30. Infection values of the top ten nodes of the nine algorithms in Ca-Astroph,** α **was set to 0.4,** *β* **was set to 1.**

| (i) EmailEU | BC | CC | DC | EC | GSI | GSM | ALSI | KBKNR | HCM |
| --- | --- | --- | --- | --- | --- | --- | --- | --- | --- |
| 1 | 1223.429 | 934.138 | 1815.212 | 1132.727 | 1709.335 | 1373.651 | 1811.546 | 1647.482 | 1561.368 |
| 2 | 10274.074 | 10101.545 | 8078.502 | 9367.215 | 8894.327 | 9198.46 | 8264.861 | 8613.61 | 9236.489 |
| 3 | 14947.683 | 14930.36 | 14764.789 | 14846.871 | 14831.236 | 14884.592 | 14803.433 | 14786.011 | 14879.464 |
| 4 | 15291.569 | 15293.867 | 15267.77 | 15282.903 | 15280.505 | 15282.384 | 15268.64 | 15276.807 | 15286.285 |
| 5 | 15325.325 | 15327.518 | 15321.907 | 15326.851 | 15327.008 | 15324.614 | 15320.965 | 15327.139 | 15329.73 |
| 6 | 15330.06 | 15333.015 | 15327.418 | 15332.973 | 15332.172 | 15329.885 | 15326.09 | 15332.758 | 15335.312 |
| 7 | 15330.796 | 15333.812 | 15328.741 | 15333.794 | 15333.157 | 15330.981 | 15327.383 | 15333.944 | 15336.364 |
| 8 | 15330.996 | 15334.025 | 15328.892 | 15334.122 | 15333.358 | 15331.195 | 15327.5 | 15334.143 | 15336.566 |
| 9 | 15331.01 | 15334.049 | 15328.952 | 15334.172 | 15333.403 | 15331.223 | 15327.541 | 15334.171 | 15336.591 |
| 10 | 15331.016 | 15334.049 | 15328.958 | 15334.194 | 15333.413 | 15331.227 | 15327.553 | 15334.171 | 15336.608 |
| 11 | 15331.016 | 15334.049 | 15328.969 | 15334.195 | 15333.413 | 15331.227 | 15327.553 | 15334.171 | 15336.608 |
| 12 | 15331.016 | 15334.049 | 15328.969 | 15334.195 | 15333.413 | 15331.227 | 15327.553 | 15334.171 | 15336.608 |
| 13 | 15331.016 | 15334.049 | 15328.969 | 15334.195 | 15333.413 | 15331.227 | 15327.553 | 15334.171 | 15336.608 |
| 14 | 15331.016 | 15334.049 | 15328.969 | 15334.195 | 15333.413 | 15331.227 | 15327.553 | 15334.171 | 15336.608 |
| 15 | 15331.016 | 15334.049 | 15328.969 | 15334.195 | 15333.413 | 15331.227 | 15327.553 | 15334.171 | 15336.608 |
| 16 | 15331.016 | 15334.049 | 15328.969 | 15334.195 | 15333.413 | 15331.227 | 15327.553 | 15334.171 | 15336.608 |
| 17 | 15331.016 | 15334.049 | 15328.969 | 15334.195 | 15333.413 | 15331.227 | 15327.553 | 15334.171 | 15336.608 |
| 18 | 15331.016 | 15334.049 | 15328.969 | 15334.195 | 15333.413 | 15331.227 | 15327.553 | 15334.171 | 15336.608 |
| 19 | 15331.016 | 15334.049 | 15328.969 | 15334.195 | 15333.413 | 15331.227 | 15327.553 | 15334.171 | 15336.608 |
| 20 | 15331.016 | 15334.049 | 15328.969 | 15334.195 | 15333.413 | 15331.227 | 15327.553 | 15334.171 | 15336.608 |
| 21 | 15331.016 | 15334.049 | 15328.969 | 15334.195 | 15333.413 | 15331.227 | 15327.553 | 15334.171 | 15336.608 |
| 22 | 15331.016 | 15334.049 | 15328.969 | 15334.195 | 15333.413 | 15331.227 | 15327.553 | 15334.171 | 15336.608 |
| 23 | 15331.016 | 15334.049 | 15328.969 | 15334.195 | 15333.413 | 15331.227 | 15327.553 | 15334.171 | 15336.608 |
| 24 | 15331.016 | 15334.049 | 15328.969 | 15334.195 | 15333.413 | 15331.227 | 15327.553 | 15334.171 | 15336.608 |
| 25 | 15331.016 | 15334.049 | 15328.969 | 15334.195 | 15333.413 | 15331.227 | 15327.553 | 15334.171 | 15336.608 |
| 26 | 15331.016 | 15334.049 | 15328.969 | 15334.195 | 15333.413 | 15331.227 | 15327.553 | 15334.171 | 15336.608 |
| 27 | 15331.016 | 15334.049 | 15328.969 | 15334.195 | 15333.413 | 15331.227 | 15327.553 | 15334.171 | 15336.608 |
| 28 | 15331.016 | 15334.049 | 15328.969 | 15334.195 | 15333.413 | 15331.227 | 15327.553 | 15334.171 | 15336.608 |
| 29 | 15331.016 | 15334.049 | 15328.969 | 15334.195 | 15333.413 | 15331.227 | 15327.553 | 15334.171 | 15336.608 |
| 30 | 15331.016 | 15334.049 | 15328.969 | 15334.195 | 15333.413 | 15331.227 | 15327.553 | 15334.171 | 15336.608 |

**Tab S31. Infection values of the top ten nodes of the nine algorithms in EmailEU,** α **was set to 0.4,** *β* **was set to 1.**

| (a) David | BC | CC | DC | EC | GSI | GSM | ALSI | KBKNR | HCM |
| --- | --- | --- | --- | --- | --- | --- | --- | --- | --- |
| 2 | 52.603 | 53.127 | 52.932 | 52.609 | 52.987 | 53.094 | 52.717 | 52.971 | 53.27 |
| 4 | 54.72 | 54.1 | 53.95 | 54.09 | 54.62 | 55.83 | 53.9 | 54.54 | 55.74 |
| 6 | 56.594 | 55.806 | 56.349 | 55.884 | 56.148 | 55.788 | 55.994 | 56.246 | 56.719 |
| 8 | 57.738 | 57.075 | 57.264 | 57.126 | 57.156 | 56.971 | 57.447 | 57.334 | 57.751 |
| 10 | 58.894 | 58.361 | 58.687 | 57.964 | 58.334 | 58.437 | 58.468 | 58.389 | 59.042 |

**Tab S32. Comparison of the numbers of activated nodes by HCM and other algorithms on David networks.**

| (b) Netscience | BC | CC | DC | EC | GSI | GSM | ALSI | KBKNR | HCM |
| --- | --- | --- | --- | --- | --- | --- | --- | --- | --- |
| 2 | 82.967 | 71.019 | 64.948 | 65.487 | 67.497 | 66.559 | 65.862 | 66.648 | 68.022 |
| 4 | 95.542 | 96.302 | 88.155 | 68.416 | 88.227 | 93.319 | 89.575 | 69.589 | 87.798 |
| 6 | 120.823 | 102.983 | 114.742 | 69.479 | 101.177 | 112.46 | 114.131 | 71.109 | 109.937 |
| 8 | 126.51 | 115.016 | 123.401 | 70.252 | 122.407 | 115.799 | 123.396 | 79.191 | 127.284 |
| 10 | 127.424 | 114.616 | 135.557 | 71.632 | 126.741 | 121.095 | 134.728 | 92.869 | 133.894 |

**Tab S33. Comparison of the numbers of activated nodes by HCM and other algorithms on Netscience networks.**

| (c) Hamsterster | BC | CC | DC | EC | GSI | GSM | ALSI | KBKNR | HCM |
| --- | --- | --- | --- | --- | --- | --- | --- | --- | --- |
| 2 | 885.6 | 885.76 | 879.91 | 878.86 | 882.7 | 881.22 | 880.87 | 876.7 | 886.88 |
| 4 | 886.2376 | 887.87 | 882.02 | 882.3366 | 885.15 | 886.28 | 883.14 | 879.77 | 888.17 |
| 6 | 890.05 | 888.32 | 885.75 | 885.54 | 887.55 | 888.24 | 885.5 | 883.18 | 891.7 |
| 8 | 892.81 | 892.5 | 892.27 | 886.79 | 890.29 | 891.69 | 889.54 | 886.37 | 893.92 |
| 10 | 897.18 | 894.38 | 896.66 | 889.74 | 894.52 | 893.98 | 891.82 | 890.42 | 898.59 |

**Tab S34. Comparison of the numbers of activated nodes by HCM and other algorithms on Hamsterster networks.**

| (d) Ca-GrQc | BC | CC | DC | EC | GSI | GSM | ALSI | KBKNR | HCM |
| --- | --- | --- | --- | --- | --- | --- | --- | --- | --- |
| 2 | 1313.248 | 1312.59 | 1315.392 | 1303.284 | 1313.802 | 1314.137 | 1317.41 | 1311.465 | 1319.137 |
| 4 | 1321.157 | 1318.275 | 1316.157 | 1317.465 | 1314.353 | 1319.332 | 1318.853 | 1313.9 | 1328.715 |
| 6 | 1339.547 | 1331.08 | 1330.05 | 1332.584 | 1328.337 | 1335.812 | 1333.495 | 1326.703 | 1344.604 |
| 8 | 1349.812 | 1344.495 | 1342.089 | 1340.198 | 1338.842 | 1345.861 | 1347.743 | 1335.931 | 1351.614 |
| 10 | 1354.509 | 1352.366 | 1351.287 | 1345.119 | 1344.95 | 1351.337 | 1350.921 | 1343.495 | 1357.82 |

**Tab S35. Comparison of the numbers of activated nodes by HCM and other algorithms on Ca-GrQc networks.**

| (e) AS | BC | CC | DC | EC | GSI | GSM | ALSI | KBKNR | HCM |
| --- | --- | --- | --- | --- | --- | --- | --- | --- | --- |
| 2 | 2386.346 | 2386.53 | 2397.665 | 2394.699 | 2397.499 | 2397.065 | 2401.802 | 2394.292 | 2410.881 |
| 4 | 2452.099 | 2464.658 | 2474.291 | 2461.457 | 2474.375 | 2470.72 | 2468.929 | 2465.99 | 2479.97 |
| 6 | 2496.802 | 2487.76 | 2508.503 | 2509.112 | 2504.127 | 2502.679 | 2502.297 | 2503.236 | 2513.663 |
| 8 | 2525.287 | 2506.88 | 2523.25 | 2527.4 | 2538.52 | 2523.32 | 2538.63 | 2519.79 | 2550.64 |
| 10 | 2551.366 | 2528.47 | 2553.65 | 2552.86 | 2560.05 | 2526.66 | 2557.18 | 2535.149 | 2569.04 |

**Tab S36. Comparison of the numbers of activated nodes by HCM and other algorithms on AS networks.**

| (f) Lastfm | BC | CC | DC | EC | GSI | GSM | ALSI | KBKNR | HCM |
| --- | --- | --- | --- | --- | --- | --- | --- | --- | --- |
| 2 | 2933.792 | 2941.51 | 2940.396 | 2939.0199 | 2930.317 | 2931.98 | 2941.149 | 2932.911 | 2943.178 |
| 4 | 2961.693 | 2963.188 | 2958.505 | 2941.109 | 2947.37 | 2946.247 | 2950.238 | 2941.812 | 2968.96 |
| 6 | 2966.499 | 2968.149 | 2968.515 | 2949.287 | 2958.029 | 2953.911 | 2976.644 | 2949.85 | 2970.812 |
| 8 | 2977.47 | 2978.693 | 2982.608 | 2956.802 | 2960.25 | 2955.366 | 2982.03 | 2959.406 | 2984.416 |
| 10 | 2992.782 | 2988.713 | 2992.366 | 2972.693 | 2969.713 | 2963.861 | 2993.931 | 2970.168 | 2995.089 |

**Tab S37. Comparison of the numbers of activated nodes by HCM and other algorithms on Lastfm networks.**

| (g) Dblp | BC | CC | DC | EC | GSI | GSM | ALSI | KBKNR | HCM |
| --- | --- | --- | --- | --- | --- | --- | --- | --- | --- |
| 2 | 4713.525 | 4709.357 | 4716.366 | 4714.842 | 4722.267 | 4719.911 | 4726.079 | 4721.564 | 4723.812 |
| 4 | 4735.514 | 4746.891 | 4745.6 | 4729.188 | 4735.416 | 4736.317 | 4746.267 | 4730.782 | 4756.287 |
| 6 | 4766.317 | 4765.386 | 4767.129 | 4745.366 | 4772.61 | 4764.85 | 4767.257 | 4760.653 | 4773.436 |
| 8 | 4775.01 | 4770.109 | 4783.228 | 4764.248 | 4780.208 | 4781.05 | 4778.01 | 4775.263 | 4788.713 |
| 10 | 4791.178 | 4785.822 | 4795.515 | 4775 | 4799.366 | 4797.248 | 4792.594 | 4788.475 | 4803.349 |

**Tab S38. Comparison of the numbers of activated nodes by HCM and other algorithms on Dblp networks.**

| (h) Ca-Astroph | BC | CC | DC | EC | GSI | GSM | ALSI | KBKNR | HCM |
| --- | --- | --- | --- | --- | --- | --- | --- | --- | --- |
| 2 | 7997.258 | 7990.733 | 7991.01 | 7996.356 | 7995.994 | 7999.733 | 8005.307 | 7995.564 | 8004.307 |
| 4 | 8009.594 | 8007.555 | 8004.693 | 7997.911 | 7998.119 | 8004.604 | 8005.347 | 8004.921 | 8010.792 |
| 6 | 8011.604 | 8008.931 | 8011.119 | 8005.119 | 8010.201 | 8007.842 | 8007.356 | 8011.792 | 8012.822 |
| 8 | 8020.753 | 8015.495 | 8012.248 | 8013.04 | 8012.455 | 8014.149 | 8012.802 | 8014.901 | 8023.03 |
| 10 | 8021.96 | 8019.386 | 8014.713 | 8015.564 | 8022.079 | 8017.495 | 8014.901 | 8020.149 | 8024.87 |

**Tab S39. Comparison of the numbers of activated nodes by HCM and other algorithms on Ca-Astroph networks.**

| (j) EmailEU | BC | CC | DC | EC | GSI | GSM | ALSI | KBKNR | HCM |
| --- | --- | --- | --- | --- | --- | --- | --- | --- | --- |
| 2 | 9297.713 | 9147.152 | 9368.436 | 9186.208 | 9336.178 | 9264.535 | 9408.556 | 9326.238 | 9390.381 |
| 4 | 9394.644 | 9229.05 | 9522.379 | 9309.086 | 9522.837 | 9401.619 | 9542.542 | 9554.389 | 9618.772 |
| 6 | 9489.97 | 9370.73 | 9594.267 | 9433.386 | 9617.802 | 9511.32 | 9613.52 | 9610.373 | 9672.109 |
| 8 | 9527.545 | 9487.762 | 9816.624 | 9514.564 | 9728.713 | 9599.644 | 9723.693 | 9717.505 | 9840.069 |
| 10 | 9633.12 | 9559.03 | 9879.663 | 9581.782 | 9800.109 | 9693.515 | 9905.376 | 9773.574 | 9937.297 |

**Tab S40. Comparison of the numbers of activated nodes by HCM and other algorithms on EmailEU networks.**
